# Active Sites‐Enriched Hierarchical Weyl Semimetal WTe_2_ Nanowire Arrays for Highly Efficient Hydrogen Evolution Reaction

**DOI:** 10.1002/advs.202500516

**Published:** 2025-04-02

**Authors:** Hyeonkyeong Kim, Youngdong Yoo

**Affiliations:** ^1^ Department of Chemistry Ajou University Suwon 16499 South Korea; ^2^ Department of Energy Systems Research Ajou University Suwon 16499 South Korea

**Keywords:** catalysis, core–shell, transition metal dichalcogenide, transition metal oxide, tungsten ditelluride, tungsten oxide, water splitting

## Abstract

Weyl semimetal tungsten ditelluride (WTe_2_), characterized by its high conductivity and robust topological surface state, possesses promising catalytic properties for electrochemical reactions. However, the synthesis of well‐defined WTe_2_ nanostructures has faced challenges, hindering their practical applications. This study introduces a new method for synthesizing Weyl semimetal WTe_2_ nanowire arrays grown vertically on conductive carbon cloth. Through a selective synthesis process, WTe_2_ and core–shell semiconductor‐semimetal WO_3−_ *_x_*–WTe_2_ nanowires are successfully fabricated via tellurization of WO_2.9_ nanowires. To gain a comprehensive understanding of the structural, chemical, and catalytic properties of these nanowires, WO_2.9_ nanowires are gradually converted to WO_3−_ *_x_*–WTe_2_ and WTe_2_ nanowires. The hierarchical structure of the WTe_2_ nanowires greatly increases the number of active sites and promotes efficient charge transfer, resulting in exceptional electrochemical catalytic performance. In the hydrogen evolution reaction, WTe_2_ nanowire arrays exhibit an exceptionally low Tafel slope of 49 mV dec^−1^, as well as remarkable stability under both high and low current densities. These exceptional properties highlight the potential of WTe_2_ nanowire arrays as highly effective electrochemical catalysts. It is expected that this facile synthesis approach will pave the way for the fabrication of well‐structured Weyl semimetal nanowires, enabling further exploration of their intriguing properties and promising applications.

## Graphical abstract

Weyl semimetal WTe_2_ possesses high conductivity and robust topological surface states. A controlled synthesis method for carbon fiber‐supported WTe_2_ and core–shell WO_3−_ *_x_*–WTe_2_ nanowire arrays is proposed. The WTe_2_ nanowires are highly crystalline, uniform, and vertically aligned, providing abundant active sites and durability in hydrogen evolution reaction. This study presents a strategy for fabricating well‐structured Weyl semimetals as electrochemical catalysts.

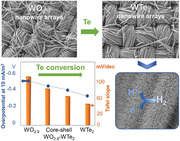

## 1 Introduction

Weyl semimetals, characterized by their unique electronic, catalytic, and quantum properties stemming from their nontrivial band structures, present exciting prospects in the fields of energy conversion and storage, electronics, and spintronics.^[^ ^1^, ^2^, ^3^, ^4^, ^5^, ^6^, ^7^ ^]^ Notably, Weyl semimetals hold promise for exceptional catalytic activity and stability in hydrogen evolution reactions (HERs), which play a crucial role in the pursuit of sustainable energy.^[^ ^8^, ^9^ ^]^ Due to their unique bulk electronic structures and nontrivial surface states, topological Weyl semimetals possess advantages as HER catalysts. The linear band crossing around the Fermi level in topological Weyl semimetals endows them with high electron mobility at room temperature, facilitating efficient charge transport kinetics during electrochemical reactions.^[^ ^10^ ^]^ In addition, Rajamathi et al. experimentally demonstrated that, in contrast to trivial metals, Weyl semimetals exhibit high HER catalytic activity despite their relatively unfavorable Gibbs free energy for hydrogen adsorption.^[^ ^8^ ^]^ Furthermore, Weyl semimetals possess topological protection, rendering them robust to surface deformations and defects caused by dangling bonds, vacancies, or doping.^[^ ^11^ ^]^ This topological robustness ensures that Weyl semimetals effectively maintain stable catalytic performance by preserving their active sites during repeated electrochemical reactions. Promising Weyl semimetal catalysts, such as those belonging to the TaAs family (NbP, NbAs, TaP, and TaAs), NbIrTe_4_, NiSi, and VAl_3_, have demonstrated excellent activity in HER.^[^ ^8^, ^12^, ^13^, ^14^ ^]^ However, in the design of highly efficient electrochemical catalysts, it is crucial to consider not only the catalyst material but also its structure.

The structure of the catalyst exerts a considerable influence on its surface area, exposure to the electrolyte, and the release of bubbles on its surface, thereby impacting the number of active sites and the charge transport path between the catalyst and the conductive substrate.^[^ ^15^ ^]^ For instance, one‐dimensional (1D) structures with linear geometry possess large surface areas, abundant exposed active sites, and rapid charge transfer pathways. This has led to the widespread adoption of 1D synthesis strategies to enhance catalyst performance.^[^ ^16^, ^17^ ^]^ Consequently, controlling the catalyst structure is essential for optimizing its catalytic activity. However, the majority of reported Weyl semimetal catalysts have been synthesized with uncontrolled structures.

The layered materials facilitate the stable formation of various low‐dimensional architectures due to their structural characteristics, offering ample active sites for catalytic activity. Tungsten ditelluride (WTe_2_) crystallizing in the orthorhombic T_d_ phase, a member of the layered transition metal dichalcogenide (TMD) family, is classified as a type‐II Weyl semimetal.^[^ ^1^, ^18^ ^]^ Layered TMDs have been shown to form quasi‐1D nanostructures such as nanowires (NWs), nanotubes, and nanorods. Quasi‐1D TMDs exhibit advantageous catalytic properties, including enhanced conductivity and increased surface area, owing to their linear structure and reduced dimensionality.^[^ ^19^, ^20^, ^21^ ^]^ Experimental studies have demonstrated that quasi‐1D group VI TMDs based on sulfur or selenium exhibit superior electrochemical catalytic performance compared to their two‐dimensional (2D) or bulk counterparts.^[^ ^22^, ^23^ ^]^ However, the exposed surfaces of semiconducting S‐ or Se‐based quasi‐1D TMDs are predominantly composed of inert basal planes, similar to their 2D counterparts, resulting in a limited number of active sites.^[^ ^24^, ^25^ ^]^ In contrast, semimetallic WTe_2_ exhibits catalytic activity not only at edge sites and defects but also on the basal plane, indicating that quasi‐1D WTe_2_ provides abundant active sites, which can enhance its catalytic performance.^[^ ^26^, ^27^ ^]^ Li et al. synthesized WTe_2_ nanoribbons using tungsten oxide prepared through hydrothermal synthesis, demonstrating their excellent performance as an HER catalyst.^[^ ^28^ ^]^ However, the catalytic performance of WTe_2_ has primarily been evaluated using exfoliated flakes owing to the challenges associated with achieving structure‐controlled growth.^[^ ^29^, ^30^ ^]^ Direct synthesis of WTe_2_ remains challenging owing to the low reactivity of tungsten and tellurium elements, the high melting point of W precursors, and the low decomposition temperature of WTe_2_.^[^ ^31^, ^32^, ^33^, ^34^ ^]^ The development of controlled growth methods is essential to effectively utilize the potential of Weyl semimetals, such as WTe_2_, for various applications, including electrochemical catalysts, topological electronics, and spintronics.^[^ ^35^, ^36^, ^37^ ^]^

In this work, we introduce a two‐step synthesis method for fabricating WTe_2_ NWs and core–shell WO_3−_ *_x_*–WTe_2_ NWs using WO_2.9_ NWs directly grown on various substrates. The highly crystalline WTe_2_ NW arrays supported on carbon fibers are uniformly and vertically aligned, providing a high density of active sites. Moreover, the direct growth on a conductive substrate ensures strong adhesion between the WTe_2_ catalysts and the substrate, resulting in good electrical and physical contact and high durability under continuous operations. WTe_2_ NW arrays demonstrated an excellent Tafel slope of 49 mV dec^−1^ and good stability at both industrial‐scale current density (500 mA cm^−2^) and low current density (20 mA cm^−2^). Our findings present a facile approach for fabricating Weyl semimetal NW arrays with exceptional catalytic, physical, and chemical properties, highlighting their potential as high‐performance electrochemical catalysts.

## 2 Results and Discussion

**Figure 1:**
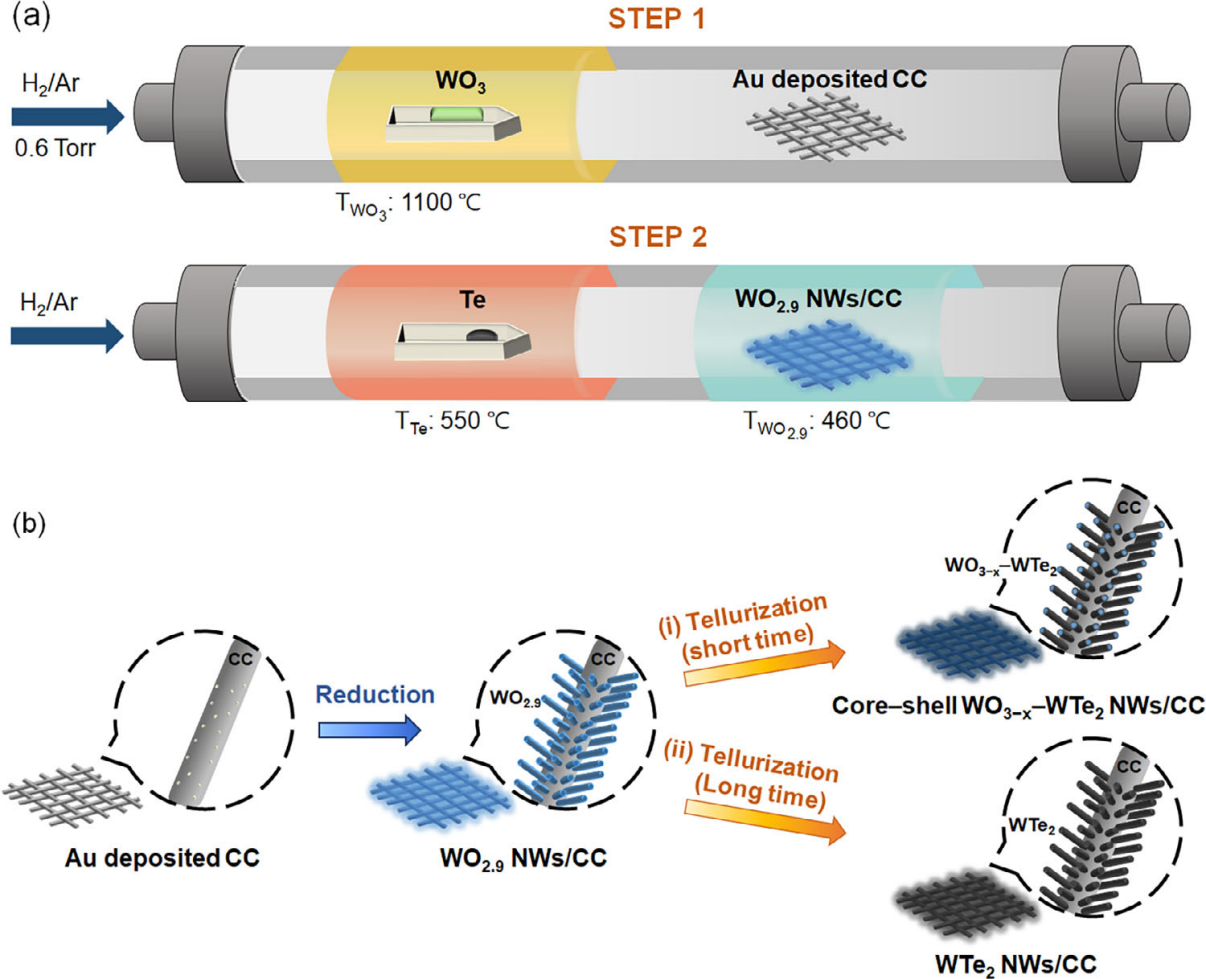
a) Schematic of the experimental setup for synthesizing WTe_2_ NW arrays and core–shell WO_3−_ *_x_*–WTe_2_ NW arrays via a two‐step synthesis process. b) Growth pathways of WO_3−_ *_x_*–WTe_2_ NWs and WTe_2_ NWs on CC.

**Figure** **1** shows the two‐step growth processes used to create 3D vertically aligned WTe_2_, core–shell WO_3−_ *_x_*–WTe_2_, and WO_2.9_ NW arrays on carbon cloth (CC) substrates. Through the partial reduction of WO_3_ by hydrogen gas, vertically aligned WO_2.9_ NW arrays were initially grown directly on CC with gold catalysts. Tungsten trioxide has an infinite array of corner‐sharing WO_6_ octahedral units in a cubic perovskite‐like structure (ReO_3_) (Figure 4g).^[^ ^38^, ^39^, ^40^ ^]^ In contrast, oxygen‐deficient tungsten sub‐oxide (WO_3−_ *_x_*) exhibits 1D anisotropy growth owing to defect‐induced surface energy variances.^[^ ^41^, ^42^, ^43^ ^]^ Te was subsequently introduced to the synthesized WO_2.9_ NWs/CC at high temperature to transform it into WTe_2_. After tellurization, the WO_2.9_ NWs acted as templates and retained their NW structure. By diffusion, the Te conversion of NWs undergoes a layer‐by‐layer transformation from WO_2.9_ to WTe_2_. Vertically aligned core–shell WO_3−_ *_x_*–WTe_2_ NW arrays [Figure 1b (i)] and WTe_2_ NW arrays [Figure 1b (ii)] were selectively produced by adjusting the tellurization time.

**Figure 2:**
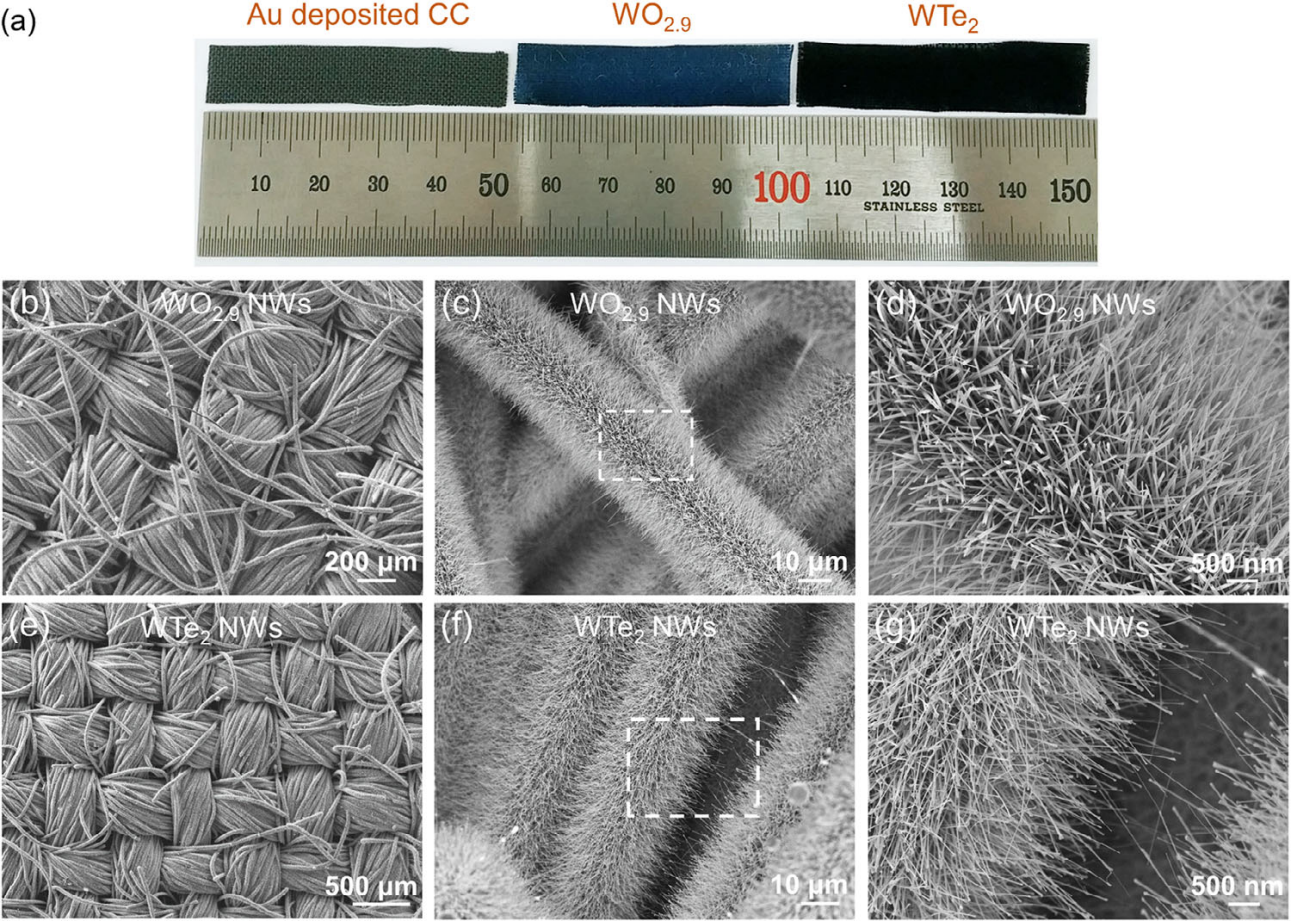
a) Optical image of the Au‐deposited CC, WO_2.9_ NWs/CC, and WTe_2_ NWs/CC. b,c) Low‐magnification SEM images of WO_2.9_ NW arrays. d) High‐magnification SEM image of the white square region indicated in (c). e,f) Low‐magnification SEM images of WTe_2_ NW arrays. g) High‐magnification SEM image of the white square region indicated in (f).

Using CC as a substrate, we successfully fabricated NW arrays across an expansive surface area (**Figure** **2**). The CC is commonly used in electrochemical applications owing to its high conductivity, robust physical properties, and flexibility.^[^ ^44^, ^45^ ^]^ As seen in Figure 2a, an optical image of Au‐deposited CC, WO_2.9_ NWs/CC, and WTe_2_ NWs/CC reveals a uniform distribution of color across substrates measuring 1 × 5 cm^2^. The homogeneous dark blue and black colors of WO_2.9_ and WTe_2_, respectively, indicate their even growth on these centimeter‐sized substrates. The ultraviolet–visible absorption spectra of WO_2.9_ NWs and WTe_2_ NWs grown directly on quartz wafers are depicted in Figure S1 (Supporting Information). The dark blue WO_2.9_ exhibits pronounced optical absorption in the short wavelength range (<400 nm), aligning with the known bandgap of tungsten suboxides, which ranges from 2.4 to 3.0 eV.^[^ ^46^, ^47^ ^]^ In contrast, the black semimetal WTe_2_ demonstrates broad absorption in the range of 200–1000 nm.

Scanning electron microscopy (SEM) was used to ascertain the morphology of the synthesized WO_2.9_ and WTe_2_ (Figure 2b–g). SEM images of bare CC and Au‐deposited CC are provided in Figure S2 (Supporting Information). The CC substrate comprises interconnected carbon fibers with a uniform diameter of ≈10 µm. As depicted in Figure 2b–d, the vertically aligned WO_2.9_ NW arrays exhibit a uniform length exceeding 10 µm and a high density on the 3D woven CC. In contrast, WO_2.9_ NWs are synthesized with relatively low density on a bare CC without Au nanoparticles (Figure S3, Supporting Information). Figure 2e–g reveals WTe_2_ NW arrays grown uniformly in a vertical orientation relative to the substrate. Owing to the high stability of WO_2.9_ NWs directly synthesized on the substrate, NWs maintain a high‐density and vertical direction after the tellurization process. Figure S4 (Supporting Information) shows core–shell WO_3−_ *_x_*–WTe_2_ NW arrays with high density and uniform NW shape synthesized under a short tellurization for 30 min. In addition, we demonstrated the versatility of the growth method by synthesizing WO_2.9_ and WTe_2_ NWs on a range of substrates, including silicon, quartz, mica, c‐cut sapphire, a‐cut sapphire, r‐cut sapphire, and m‐cut sapphire (Figures S5 and S6, Supporting Information).

**Figure 3:**
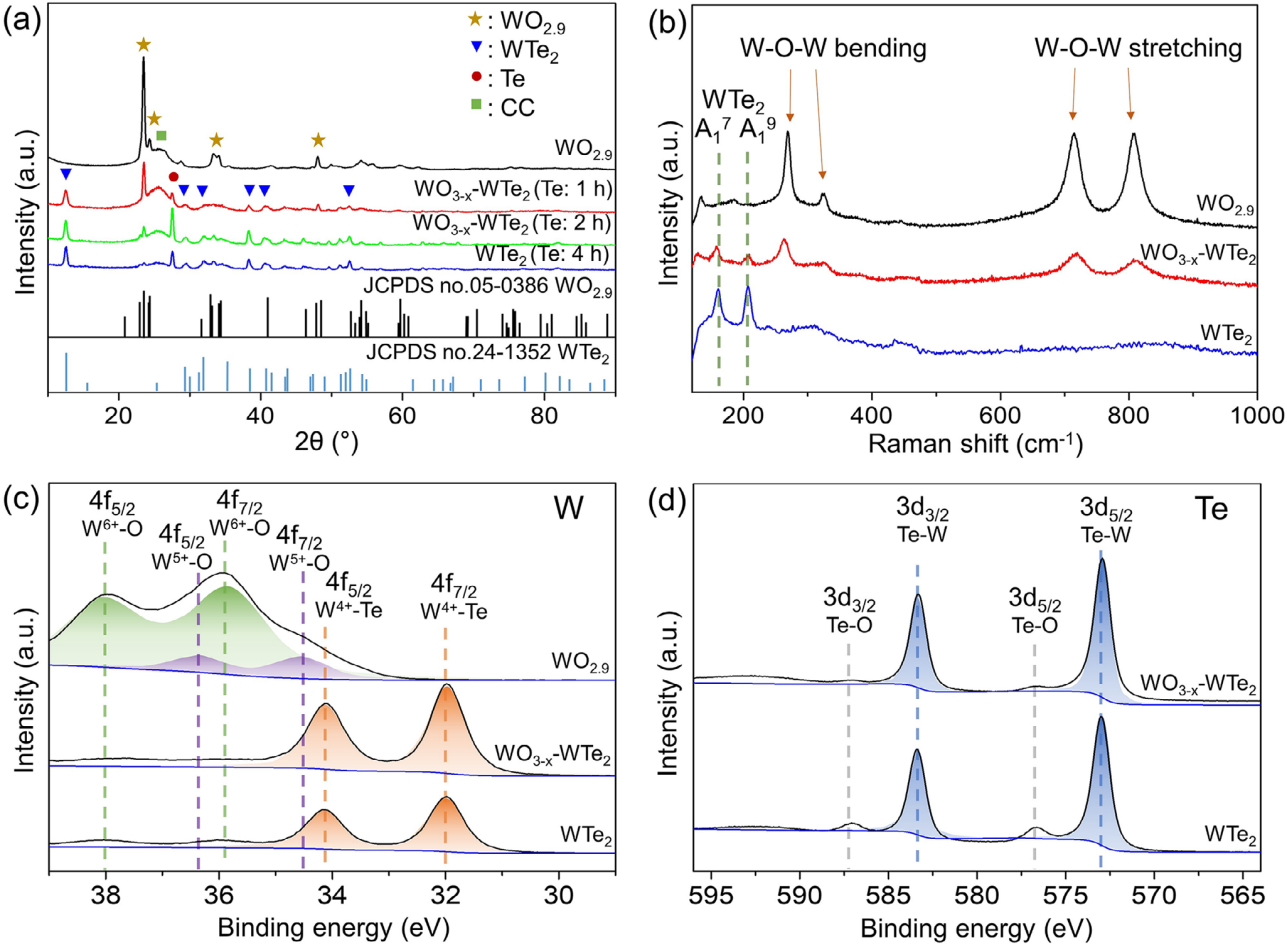
a) XRD patterns and b) Raman spectra of WO_2.9_ NWs, core–shell WO_3−_ *_x_*–WTe_2_ NWs, and WTe_2_ NWs. XPS spectra for c) W 4f and d) Te 3d regions of the as‐synthesized NWs.

The properties of NWs transformed from WO_2.9_ to WTe_2_ via tellurization were corroborated through X‐ray diffraction (XRD), Raman, and X‐ray photoelectron spectroscopy (XPS) measurements (**Figure** **3**). Figure 3a shows the XRD patterns of as‐synthesized WO_2.9_, core–shell WO_3−_ *_x_*–WTe_2_, and WTe_2_ NWs. The XRD pattern of WO_2.9_ NWs corresponds to a monoclinic WO_2.9_ crystal structure (JCPDS card no. 05‐0386), with the primary diffraction peaks located at 2θ = 23.5°, 24.4°, 34.1°, and 48.1° corresponding to the (010), (106), (116), and (020) planes, respectively (denoted by asterisks in Figure 3a). The XRD patterns of core–shell WO_3−_ *_x_*–WTe_2_ NWs obtained after tellurization for 1 and 2 h exhibit peaks corresponding to WO_2.9_, WTe_2_, and Te. In addition to the diffraction peaks of WO_2.9_, additional peaks are observed at 12.5°, 29.2°, 32.0°, 38.4°, 40.6°, and 52.5°, which can be attributed to the (002), (021), (103), (024), (122), and (200) planes of orthorhombic WTe_2_ (JCPDS card no. 24‐1352; denoted by triangles in Figure 3a). Also, a peak at 27.6° corresponds to the (101) plane of Te (denoted by a circle in Figure 3a according to JCPDS card no. 36‐1452). The presence of these peaks confirms the coexistence of WO_2.9_ and WTe_2_ in the core–shell WO_3−_ *_x_*–WTe_2_ structure. Furthermore, the intensity of the WTe_2_ peak at 12.5° increases relative to the WO_2.9_ peak at 23.5° as the tellurization time is extended. These observations indicate that WO_2.9_ is partially converted to WTe_2_ during the initial stages of tellurization and that this conversion progresses further with increasing tellurization duration. The sharp peaks of WO_2.9_, WO_3−_ *_x_*–WTe_2_, and WTe_2_ NWs attest to the high crystallinity of the synthesized materials. In contrast, the XRD pattern of the WTe_2_ NWs obtained after 4 h of tellurization exhibits only diffraction peaks corresponding to WTe_2_ and Te. The absence of the WO_2.9_ peak confirms the complete conversion of WO_2.9_ NWs to WTe_2_ NWs. The Te diffraction peak appears not only in the WO_3−_ *_x_*–WTe_2_ NWs synthesized through short tellurization processes of 1 and 2 h (Figure 3a) but also in the WO_3−_ *_x_*–WTe_2_ NWs synthesized through tellurization process at a low Te temperature of 500 °C (Figure S7, Supporting Information). We believe that the Te diffraction peak originates from unreacted Te particles deposited on the CC substrate.

Raman spectroscopy measurements were performed on the synthesized WO_2.9_, WO_3−_ *_x_*–WTe_2_, and WTe_2_ NWs using a 532 nm laser source. The Raman spectrum of WO_2.9_ NWs exhibits peaks at 270 and 326 cm^−1^, which correspond to the bending vibration of the W─O─W bond, and peaks at 712 and 808 cm^−1^, which correspond to the stretching vibration of the W─O─W bond (Figure 3b).^[^ ^48^ ^]^ The Raman spectrum of core–shell WO_3−_ *_x_*–WTe_2_ NWs obtained after tellurization of WO_2.9_ NWs for 1 h reveals peaks corresponding to both WO_2.9_ and WTe_2_. The peaks at 161 and 208 cm^−1^ in the Raman spectrum of core–shell WO_3−_ *_x_*–WTe_2_ correspond to the A_1_^7^ and A_1_^9^ modes of WTe_2_.^[^ ^49^, ^50^ ^]^ The Raman spectrum of WTe_2_ obtained via tellurization of WO_2.9_ NWs for 4 h exhibits only the A_1_^7^ and A_1_^9^ modes of WTe_2_.

XPS measurements were performed to investigate the surface composition and chemical states of the as‐synthesized WO_2.9_, core–shell WO_3−_ *_x_*–WTe_2_, and WTe_2_ NWs surfaces (Figure 3c,d; Figures S8 and S9, Supporting Information). The XPS survey results of WO_2.9_, WO_3_, WO_3−_ *_x_*–WTe_2_, and WTe_2_ NWs on CC are presented in Figure S8a (Supporting Information). WO_2.9_/CC and WO_3_/CC exhibit XPS signals of W, O, Au, and C elements, whereas WTe_2_/CC and WO_3−_ *_x_*–WTe_2_/CC exhibit those of W, O, C, and Te elements. The W 4f spectrum of WO_2.9_ reveals peaks at 38.1 and 35.8 eV, corresponding to the W 4f_5/2_ and W 4f_7/2_ levels for W^6+^, respectively, and peaks at 36.5 and 34.5 eV, corresponding to the W 4f_5/2_ and W 4f_7/2_ levels for W^5+^, respectively.^[^ ^51^ ^]^ The presence of W^5+^ binding energy peaks in the W 4f spectra of WO_2.9_ indicates oxygen vacancies in WO_3_. Unlike WO_2.9_, the W 4f spectrum of WO_3_ exhibits only two pronounced peaks corresponding to W^6+^ (Figure S8b, Supporting Information).^[^ ^48^, ^51^, ^52^ ^]^ The W peaks of WO_3−_ *_x_*–WTe_2_ and WTe_2_ shift to lower binding energies at 34.1 and 32.0 eV, corresponding to the W 4f_5/2_ and W 4f_7/2_ levels for W^4+^, respectively. The Te 3d spectra of core–shell WO_3−_ *_x_*–WTe_2_ and WTe_2_ reveal two prominent peaks related to W─Te bonding at 583.3 eV (Te 3d_3/2_) and 572.9 eV (Te 3d_5/2_).^[^ ^31^, ^50^ ^]^ Two minor peaks corresponding to Te─O located at 587.4 and 577.0 eV were also detected. The appearance of Te─O peaks in the XPS measurements of WTe_2_ and WO_3−_ *_x_*–WTe_2_ NWs could be attributed to the partial oxidation of the WTe_2_ surface. In surface‐sensitive XPS measurements, core–shell WO_3−_ *_x_*–WTe_2_ NWs exhibit only the peaks of the WTe_2_ shell without the peaks of the WO_3−_ *_x_* core.^[^ ^53^ ^]^ Figure S9 (Supporting Information) shows that Au element was detected in WO_2.9_ NWs, whereas Au signals were not observed in WO_3−_ *_x_*–WTe_2_ (tellurization: 1 h) and WTe_2_ NWs. This suggests that the Au was removed from the NWs during the tellurization process.

**Figure 4:**
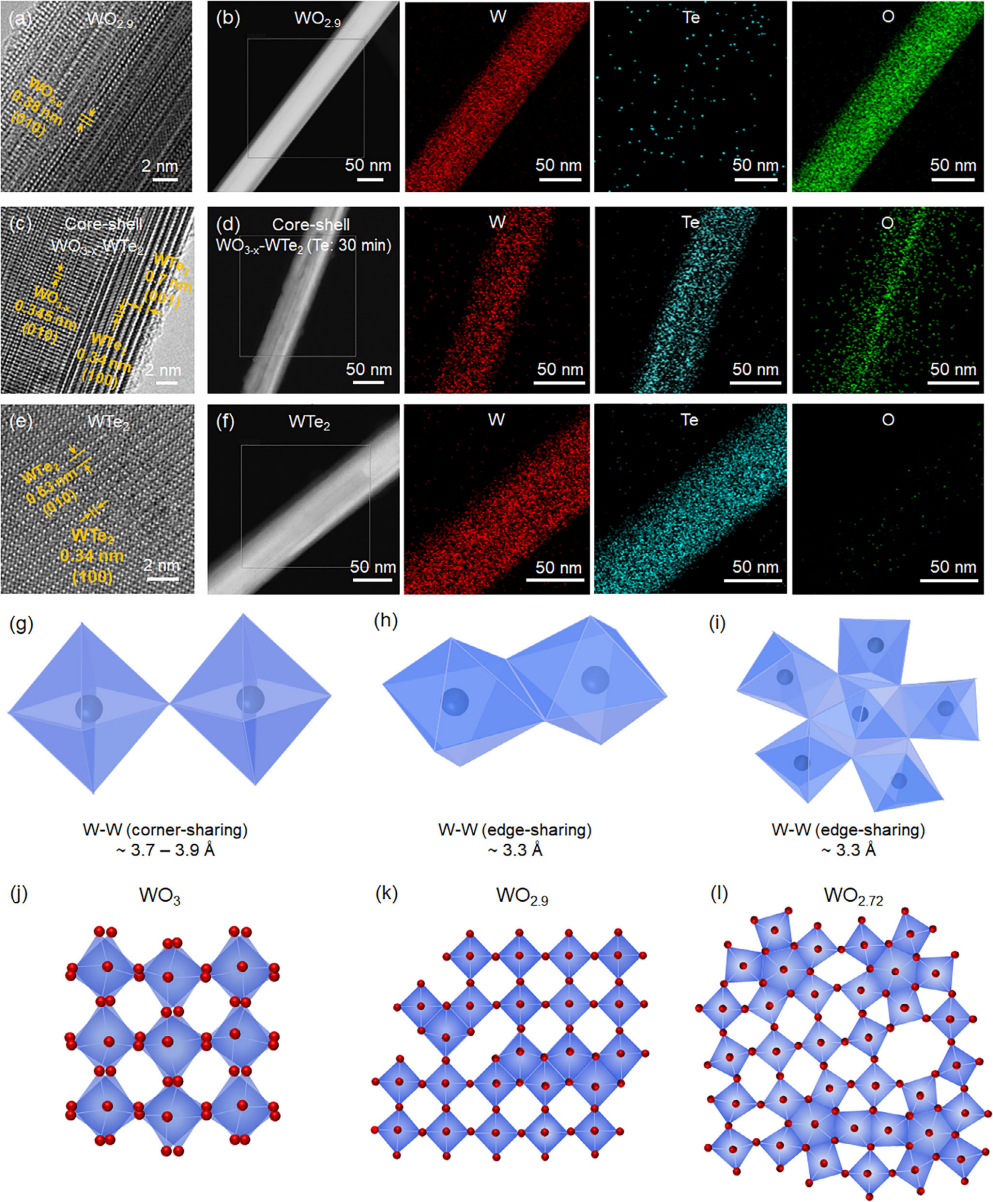
TEM images, STEM images, and EDS element maps of a,b) WO_2.9_ NWs, c,d) core–shell WO_3−_ *_x_*–WTe_2_ NWs (tellurization for 30 min), and e,f) WTe_2_ NWs, respectively. The structures of tungsten oxide compounds. The W─W distances g) between two corner‐sharing [WO_6_]‐octahedra, h) between two edge‐sharing [WO_6_]‐octahedra, and i) between a [WO_6_]‐octahedra and a bipyramidal pentagonal column. j–l) The structures of monoclinic WO_3_, WO_2.9_, and WO_2.72_. W is shown in blue, O in red.

To elucidate the structural transformations in NWs induced by tellurization at the atomic level, high‐resolution transmission electron microscopy (HRTEM) imaging and energy‐dispersive spectroscopy (EDS) mapping analysis were performed on WO_2.9_ NWs, core–shell WO_3−_ *_x_*–WTe_2_, and WTe_2_ NWs (**Figure** **4**). The lattice spacing of 0.38 nm observed in WO_2.9_ corresponds to the spacing of the (010) planes of monoclinic WO_2.9_ (Figure 4a and Figure S10, Supporting Information).^[^ ^54^ ^]^ EDS mapping of the WO_2.9_ NW, ≈50 nm in thickness, reveals a uniform distribution of W and O elements (Figure 4b).

The HRTEM images of WO_3−_ *_x_*–WTe_2_ NWs synthesized via tellurization for 30 min reveal a core–shell structure comprising a core of WO_3−x_ and a shell of WTe_2_ (Figure 4c,d and Figure S11, Supporting Information). The HRTEM image of the WO_3−_ *_x_* core of the WO_3−_ *_x_*–WTe_2_ NW exhibits the lattice spacing of 0.345 nm, corresponding to the spacing of the (100) planes of monoclinic WO_3−_ *_x_*. The HRTEM image of the WTe_2_ shell of the WO_3−_ *_x_*–WTe_2_ NW exhibits the lattice spacing of 0.34 and 0.7 nm that correspond to the spacings of the (100) and (001) planes of orthorhombic WTe_2_ (Figure 4c), respectively. The fast Fourier transformation patterns obtained from the core and shell of WO_3−_ *_x_*–WTe_2_ NWs demonstrate the high crystallinity of the core WO_3−_ *_x_* and shell WTe_2_ (Figure S10g,h, Supporting Information). The low‐ and high‐magnification EDS maps of WO_3−_ *_x_*–WTe_2_ NWs clearly depict the core–shell structure (Figure 4d and Figure S12, Supporting Information). In the EDS maps of core–shell WO_3−_ *_x_*–WTe_2_, the W element is uniformly distributed on the surface, while the O and Te elements are concentrated in the core and edge of the wires, respectively. The core–shell WO_3−_ *_x_*–WTe_2_ structure aligns with the XRD and XPS measurements presented in Figure 3. The XRD data of core–shell WO_3−_ *_x_*–WTe_2_ exhibit peaks corresponding to both WO_2.9_ and WTe_2_, whereas the XPS data obtained through surface‐sensitive measurements reveal only peaks consistent with WTe_2_. In addition, the gradual transformation from WO_3−_ *_x_* to WTe_2_ with increasing tellurization time can be observed in the TEM images of WO_3−_ *_x_*–WTe_2_ NWs processed under different tellurization durations (Figure S13, Supporting Information). The tellurization process of WO_3−_ *_x_* NWs was conducted for 3, 10, 30, and 60 min, respectively, and a progressive thickening of the WTe_2_ shell in WO_3−_ *_x_*–WTe_2_ was confirmed as the tellurization time increased.

As shown in Figure 4e, the HRTEM image of the WTe_2_ NW obtained via tellurization for 4 h exhibits the lattice spacing of 0.63 and 0.34 nm, corresponding to the spacings of the (010) and (100) planes of the orthorhombic WTe_2_, respectively.^[^ ^50^ ^]^ The EDS mapping images of the WTe_2_ clearly illustrate the uniform distribution of W and Te elements (Figure 4f). Notably, the WTe_2_ NWs and core–shell WO_3−_ *_x_*–WTe_2_ NWs fabricated through the tellurization process of WO_2.9_ NWs maintain a diameter of ≈50 nm (Figure 4b,d,f).

The conversion process from WO_2.9_ NW arrays to WTe_2_ NW arrays via tellurization proceeds as follows. As depicted in Figure 4a and Figure S10a (Supporting Information), WO_2.9_ is primarily composed of corner‐sharing octahedra with a lattice spacing of 0.38 nm despite being partially oxygen‐deficient. During the tellurization process, WO_2.9_ is reduced by Te vapor and hydrogen gas. The oxygen atoms on the surface of WO_2.9_ NWs are replaced by Te atoms to form shell WTe_2_ layers, resulting in core–shell WO_3−_ *_x_*–WTe_2_ NW structures (Figure 4c,d and Figures S10 and S12, Supporting Information). Concomitantly, the tungsten suboxide undergoes structural changes owing to the severe oxygen deficiency induced by the tellurization process. Under oxygen‐deficient conditions, a portion of the tungsten oxide undergoes a structural transformation from corner‐sharing [WO_6_] octahedra with a lattice spacing of ≈0.38 nm to edge‐sharing [WO_6_] octahedra or [WO_7_] bipyramid pentagons with a lattice spacing of 0.33 nm (Figure 4g–i).^[^ ^40^ ^]^ This transformation is driven by the reduction reaction and subsequent oxygen deficiency. The spacing of the (100) planes of the WO_3−_ *_x_* is smaller than the spacing of the (100) planes of the WO_2.9_ due to the structural changes caused by the presence of oxygen vacancies in WO_3−_ *_x_* (Figure 4c and Figure S10, Supporting Information). The oxygen‐deficiency‐induced structural change reduces the lattice mismatch between the tungsten sub‐oxide core and the WTe_2_ shell, which has a lattice spacing of 0.34 nm. This allows the NW array morphology of WO_2.9_ to be maintained after the tellurization process, leading to the stable formation of core–shell WO_3−_ *_x_*–WTe_2_ NWs and WTe_2_ NWs. When sufficient Te vapor is supplied to the NWs, Te atoms diffuse from the WTe_2_ shell into the core WO_3−_ *_x_*, eventually converting the NWs entirely into WTe_2_ NWs.

Our findings, which demonstrate the transformation of tungsten oxide NWs into high‐quality WTe₂ NWs, contrast with previous reports showing sulfurization of tungsten oxide NWs results in the formation of WS_2_ with altered structures such as nanotubes, nanomeshes, nanoflowers, or NW‐nanoflake architectures.^[^ ^25^, ^55^, ^56^, ^57^, ^58^, ^59^, ^60^ ^]^ We hypothesize that the structural differences observed in synthesized materials arise from the lattice mismatch between tungsten oxide and WS_2_. As previously mentioned, the lattice spacing of WTe₂ at the interface closely matches that of tungsten oxide. In contrast, the lattice spacing along the direction of (100) of WS₂ in contact with tungsten oxide is reported to be ≈0.27 nm, showing a significant mismatch with tungsten oxide.^[^ ^56^, ^61^ ^]^ As a result, this lattice mismatch leads to structural transformations during the sulfurization of tungsten oxide NWs into WS₂.

**Figure 5:**
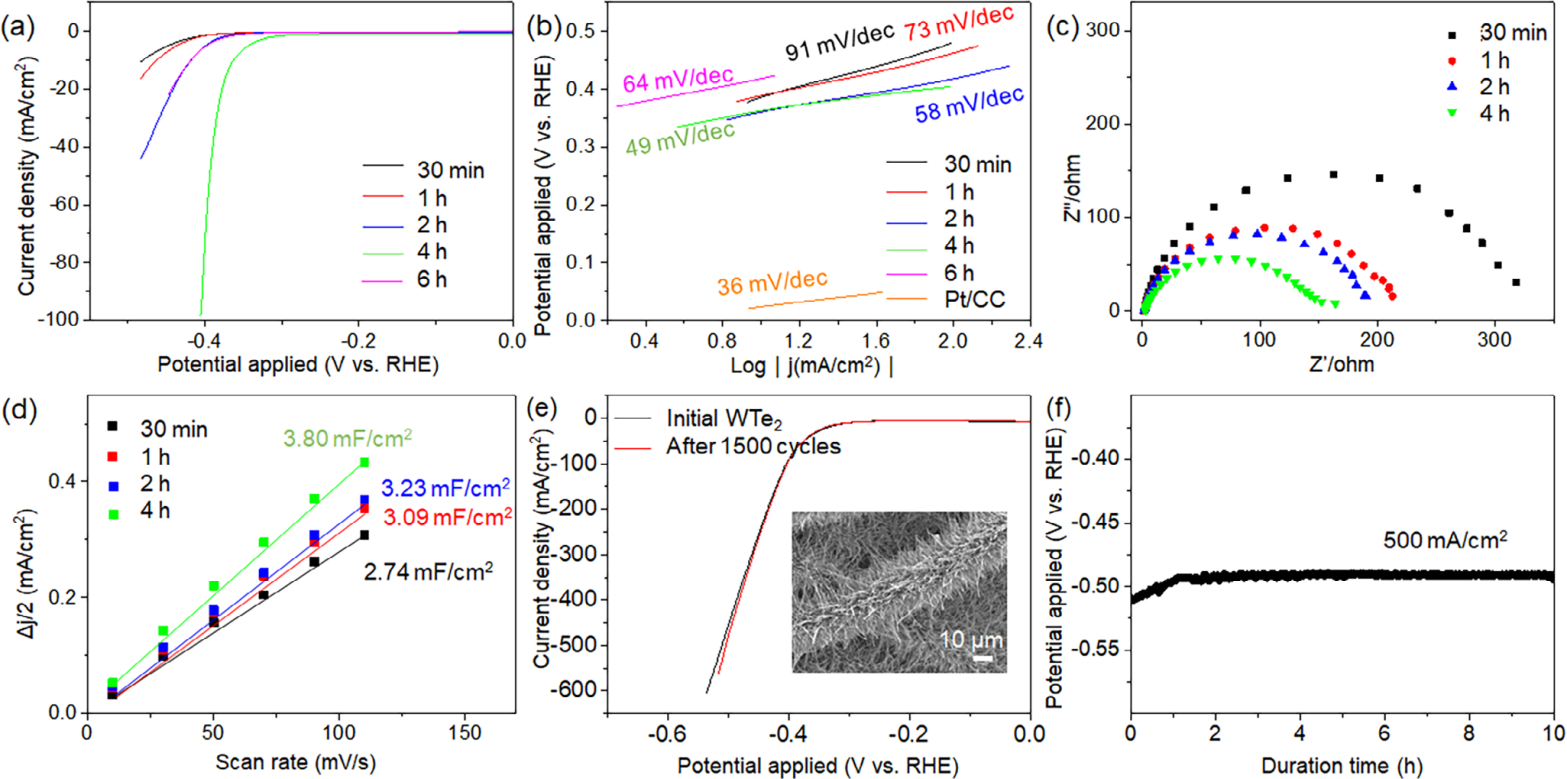
Electrochemical performance of WTe_2_ NWs and core–shell WO_3−_ *_x_*–WTe_2_ NWs at different tellurization times. a) LSV polarization curves, b) Tafel plots, c) Nyquist plots, and d) correlation between current density and scan rate for the catalysts. e) LSV polarization curves of WTe_2_ NWs before and after 1500 HER cycles, with the inset showing an SEM image of WTe_2_ NWs post‐HER. f) Stability test of WTe_2_ NWs at a current density of 500 mA cm^−2^.

We evaluated the catalytic performance of the synthesized core–shell WO_3−_ *_x_*–WTe_2_ NWs/CC and WTe_2_ NWs/CC. The core–shell WO_3−_ *_x_*–WTe_2_ NWs/CC were prepared by tellurization of WO_2.9_ NWs for varying durations of 30 min, 1 h, 2 h, and 4 h (**Figure** **5**). Electrochemical measurements were performed in a standard three‐electrode cell using 0.5 M H_2_SO_4_ electrolyte. The as‐synthesized catalysts grown on CC, a platinum wire, and Ag/AgCl served as the working electrode, counter electrode, and reference electrode, respectively. Notably, CC used as a support possesses excellent conductivity, high physical strength, and high flexibility, making it widely used as a support in electrochemical applications.^[^ ^45^ ^]^ Furthermore, the direct growth of the catalyst on the support ensures excellent electrical contact and strong physical adsorption between the catalyst and support. The polarization curves of the catalyst obtained using linear sweep voltammetry (LSV) were corrected by compensating for the ohmic drop (*iR* drop). The Tafel plots in Figure 5b and Figures S14 and S15 (Supporting Information) were derived from the LSV curves (*η* = *b* log *j* + *a*, where *η* is the overpotential, *j* is the current density, *b* is the Tafel slope, and *a* is the Tafel constant). For comparison of electrochemical performance, Pt/CC was prepared by depositing a 15–20 nm thick platinum layer on the CC. The LSV curve of the Pt/CC is shown in Figure S14 (Supporting Information).

The improvement in catalytic performance owing to the tellurization of the NWs was corroborated by a reduction in both the overpotential and Tafel slope (Figure 5a,b). The overpotential of the catalyst obtained at a current density of 10 mA cm^−2^ decreased from 0.48 V versus (reversible hydrogen electrode [RHE]) for core–shell WO_3−_ *_x_*–WTe_2_ NWs/CC (tellurization for 30 min) to 0.36 V for WTe_2_ NWs/CC. Notably, WTe_2_ with a 3D vertically aligned NW arrays exhibited a low Tafel slope of 49 mV dec^−1^, which is superior to previously reported WTe_2_‐based HER catalysts (Table S1, Supporting Information). In addition, compared to other S‐ or Se‐based TMD catalysts, WTe_2_ NWs exhibit a very low Tafel slope value (Table S2, Supporting Information). The Tafel slope value of 49 mV dec^−1^ suggests that the rate‐limiting step for WTe_2_ NWs is the Volmer–Heyrovsky step.^[^ ^62^, ^63^ ^]^ The HER mechanism is described in the Supporting Information. After Te conversion, the Tafel slope of core–shell WO_3−_ *_x_*–WTe_2_ NWs decreases from 91 mV dec^−1^ (tellurization for 30 min) to 58 mV dec^−1^ (tellurization for 2 h) (Figure 5b). For comparison, we analyzed the catalytic performance of WO_2.9_ NWs/CC and CC, showing high Tafel slopes of 123 and 315 mV dec^−1^, respectively (Figure S15, Supporting Information). Conversely, WTe_2_ NWs obtained by tellurization for 6 h have a relatively high Tafel slope of 64 mV dec^−1^, suggesting reduced catalytic activity compared to WTe_2_ obtained by tellurization for 4 h (Figure 5b). The inferior performance of the catalyst synthesized via an extended tellurization process can be attributed to the overabundance of Te particles. Figure S16 (Supporting Information) presents the SEM image and EDS results of WTe_2_ NWs obtained after tellurization for 6 h.

Electrochemical impedance spectroscopy (EIS) was performed to elucidate the charge transfer process of WTe_2_ NW and core–shell WO_3−_ *_x_*–WTe_2_ NW catalysts in HER (Figure 5c). The Nyquist plots of WTe_2_ NWs and core–shell WO_3−_ *_x_*–WTe_2_ NWs exhibit semicircles, with the charge transfer resistance (*R*_ct_) decreasing substantially as Te conversion increases. Weyl semimetal WTe_2_ NWs, possessing the lowest *R*_ct_ of 72 Ω, demonstrate superior HER catalytic activity attributed to their expedited charge transfer rate compared to semiconducting WO_2.9_ NWs (Figure S17, Supporting Information).

The catalytic performance is influenced by both the inherent activity of the catalyst and the number of active sites, which is typically assessed using electrochemical active surface area (ECSA) measurements.^[^ ^64^ ^]^ The ECSA is directly proportional to the electrochemical double double‐layer capacitance (*C*_dl_) and is estimated by performing cyclic voltammetry (CV) in the non‐Faradaic potential range with scan rates ranging from 10 to 110 mV s^−1^ (Figure S18, Supporting Information). As illustrated in Figure 5d, the *C*_dl_ values increase slightly with Te conversion. Various layered S‐ or Se‐based TMDs, such as WS_2_, MoS_2_, MoSe_2_, WS_2_, and WSe_2_ are thermodynamically stable in the semiconducting 2H phase, but their basal planes are catalytically inert, limiting active sites for HER. This limitation persists even in quasi‐1D structure because the dominant exposed surface of quasi‐1D structures is the basal plane. In contrast, semimetallic WTe_2_ demonstrates catalytic activity not only at the edge sites but also on its basal planes.^[^ ^24^, ^25^ ^]^ Thus, WTe_2_ NWs possess abundant catalytic active sites though they primarily expose the basal plane. Furthermore, 3D vertically aligned WTe_2_ NW arrays lead to the increased surface area, further improving catalytic performance.

For industrial applications of electrochemical catalytic water splitting, HER catalysts must operate at high current densities (at least 500 mA cm^−2^).^[^ ^65^ ^]^ However, the stability and performance of the Weyl semimetal WTe_2_ have primarily been assessed at lower current densities, below 100 mA cm^−2^.^[^ ^28^, ^66^, ^67^, ^68^ ^]^ We demonstrated the remarkable operational stability of WTe_2_ NWs/CC by operating it for 1500 continuous cycles in a potential range of −0.54 to 0.2 V, successfully achieving a current density that exceeded 500 mA cm^−2^ (Figure 5e). The LSV curve of WTe_2_/CC after 1500 cycles showed no considerable decline compared to pristine WTe_2_/CC. The WTe_2_ NWs synthesized directly on the CC remain vertically aligned in three‐dimensional formation even after extended HER use (Figure 5e and Figure S19, Supporting Information). The roughened surface of the NWs following long‐term HER is attributed to the dissolution of Te in the acidic environment (WTe_2_ → WTe_2−_ *_x_* + *x*Te).^[^ ^68^ ^]^ Despite the surface deformation, WTe_2_ has maintained its excellent catalytic performance owing to the inherent robustness of the Weyl semimetal against surface defects. In addition, galvanostatic measurement of WTe_2_ NWs/CC demonstrates durability, as indicated by the potential of ≈−0.5 V required to achieve a current density of 500 mA cm^−2^ during continuous 10 h operation (Figure 5f). Furthermore, the WTe_2_ NWs/CC exhibit good operational stability in continuous HER at a low current density of 20 mA cm^−2^ at −0.38 V during continuous 12 h operation (Figure S20, Supporting Information). The high electrochemical stability of WTe_2_/CC is attributed to the unique surface states of the topological semimetal and the strong adhesion between catalysts and the substrate resulting from direct synthesis. The unique surface states of the topological materials are expected to provide not only high surface carrier densities but also highly robust surfaces against various disruptions, such as defects, morphological changes, and contamination.^[^ ^8^, ^9^, ^11^ ^]^ This protection of surface states in topological materials, which arises from specific symmetries and bulk states, ensures that the existing active sites remain stable for the overall catalyst performance, even in the presence of potential disturbances that could undermine the effectiveness of the catalysts.^[^ ^4^ ^]^ Furthermore, we directly synthesized WTe_2_ NWs on conductive CC. The direct growth of WTe_2_ NWs not only enables the formation of vertically aligned three‐dimensional structures with abundant active sites but also provides strong physical adhesion between WTe_2_ NWs and CC. As a result, WTe_2_/CC possesses mechanical stability, minimizes the separation and desorption of WTe_2_ catalysts from CC substrates, and prevents the agglomeration of WTe_2_ catalyst during long‐term catalytic reactions.

## 3 Conclusion

In summary, we successfully synthesized vertically aligned arrays of Weyl semimetal WTe_2_ NWs to create an efficient catalyst for hydrogen production. By directly growing WO_2.9_ NWs on substrates, we selectively synthesized WTe_2_ NWs and WO_3−_ *_x_*–WTe_2_ NWs by controlling the tellurization time. These NWs, supported on 3D woven carbon fibers, form a uniform, high‐density, vertically aligned structure that provides a high density of active sites and excellent operational durability. Hierarchically structured WTe_2_ NW arrays demonstrated exceptional HER performance, featuring a low Tafel slope of 49 mV dec^−1^ and remarkable catalytic stability across both high and low current densities. We fabricated well‐structured Weyl semimetal NW arrays and investigated their potential in electrochemical catalysis, offering valuable insights into the applicability of Weyl semimetals and TMD NWs for fundamental research and technological applications.

## Conflict of Interest

The authors declare no conflict of interest.

## Supporting information



Supporting Information

## Data Availability

The data that support the findings of this study are available from the corresponding author upon reasonable request.

## References

[1] A. A. Soluyanov , D. Gresch , Z. Wang , Q. Wu , M. Troyer , X. Dai , B. A. Bernevig , Nature 2015, 527, 495. 26607545 10.1038/nature15768

[2] S. Jia , S.‐Y. Xu , M. Z. Hasan , Nat. Mater. 2016, 15, 1140. 27777402 10.1038/nmat4787

[3] B. Yan , C. Felser , Annu. Rev. Condens. Matter Phys. 2017, 8, 337.

[4] R. Xie , T. Zhang , H. Weng , G.‐L. Chai , Small Sci. 2022, 2, 2100106. 40212671 10.1002/smsc.202100106 PMC11935991

[5] C. Guo , V. S. Asadchy , B. Zhao , S. Fan , eLight 2023, 3, 2.

[6] Q. Wang , J. Li , J. Besbas , C.‐H. Hsu , K. Cai , L. Yang , S. Cheng , Y. Wu , W. Zhang , K. Wang , T.‐R. Chang , H. Lin , H. Chang , H. Yang , Adv. Sci. 2018, 5, 1700912. 10.1002/advs.201700912 PMC6010885 29938171

[7] C. Gong , Y. Zhang , W. Chen , J. Chu , T. Lei , J. Pu , L. Dai , C. Wu , Y. Cheng , T. Zhai , L. Li , J. Xiong , Adv. Sci. 2017, 4, 1700231. 10.1002/advs.201700231 PMC5737141 29270337

[8] C. R. Rajamathi , U. Gupta , N. Kumar , H. Yang , Y. Sun , V. Süß , C. Shekhar , M. Schmidt , H. Blumtritt , P. Werner , B. Yan , S. Parkin , C. Felser , C. N. R. Rao , Adv. Mater. 2017, 29, 1606202. 10.1002/adma.201606202 28295640

[9] U. Gupta , C. R. Rajamathi , N. Kumar , G. Li , Y. Sun , C. Shekhar , C. Felser , C. N. R. Rao , Dalton Trans. 2020, 49, 3398. 32129389 10.1039/d0dt00050g

[10] D.‐X. Liu , H. Hong , Q. Cao , D. Wang , Y. Du , ChemPhysChem 2024, 25, s202300942. 10.1002/cphc.202300942 38270388

[11] X. Zhang , L. Wang , M. Li , W. Meng , Y. Liu , X. Dai , G. Liu , Y. Gu , J. Liu , L. Kou , Mater. Today 2023, 67, 23.

[12] M. Samanta , H. Tan , S. Laha , Vignolo‐González , H. Alejandro , L. Grunenberg , S. Bette , V. Duppel , P. Schützendübe , A. Gouder , B. Yan , B. V. Lotsch , Adv. Energy Mater. 2023, 13, 2300503.

[13] W. Liu , X. Zhang , W. Meng , Y. Liu , X. Dai , G. Liu , iScience 2022, 25, 103543. 34977505 10.1016/j.isci.2021.103543 PMC8683596

[14] X.‐P. Kong , T. Jiang , J. Gao , X. Shi , J. Shao , Y. Yuan , H.‐J. Qiu , W. Zhao , J. Phys. Chem. Lett. 2021, 12, 3740. 33844544 10.1021/acs.jpclett.1c00238

[15] Q. Fu , J. Han , X. Wang , P. Xu , T. Yao , J. Zhong , W. Zhong , S. Liu , T. Gao , Z. Zhang , L. Xu , B. Song , Adv. Mater. 2021, 33, 1907818. 32578254 10.1002/adma.201907818 PMC11468112

[16] L. Zhang , H. Zhao , S. Xu , Q. Liu , T. Li , Y. Luo , S. Gao , X. Shi , A. M. Asiri , X. Sun , Small Struct. 2021, 2, 2000048.

[17] Q. Zeng , N. Deng , S. Wang , S. Luo , G. Wang , H. Gao , Y. Li , H. Wang , B. Cheng , W. Kang , ChemElectroChem 2022, 9, 202200946.

[18] P. Li , Y. Wen , X. He , Q. Zhang , C. Xia , Z.‐M. Yu , S. A. Yang , Z. Zhu , H. N. Alshareef , X.‐X. Zhang , Nat. Commun. 2017, 8, 2150. 29247186 10.1038/s41467-017-02237-1 PMC5732285

[19] F. Qin , T. Ideue , W. Shi , X.‐X. Zhang , M. Yoshida , A. Zak , R. Tenne , T. Kikitsu , D. Inoue , D. Hashizume , Y. Iwasa , Nano Lett. 2018, 18, 6789. 30285446 10.1021/acs.nanolett.8b02647

[20] B. Kim , N. Park , J. Kim , Nat. Commun. 2022, 13, 3237. 35688833 10.1038/s41467-022-31018-8 PMC9187746

[21] M. Bar‐Saden , R. Tenne , Nat. Mater. 2024, 23, 310. 37443380 10.1038/s41563-023-01609-x

[22] K. Xu , F. Wang , Z. Wang , X. Zhan , Q. Wang , Z. Cheng , M. Safdar , J. He , ACS Nano 2014, 8, 8468. 25110810 10.1021/nn503027k

[23] M. B. Sreedhara , Y. Miroshnikov , K. Zheng , L. Houben , S. Hettler , R. Arenal , I. Pinkas , S. S. Sinha , I. E. Castelli , R. Tenne , J. Am. Chem. Soc. 2022, 144, 10530. 35656885 10.1021/jacs.2c03187 PMC9204813

[24] E. Magee , F. Tang , E. Ozdemir , M. Walker , T. Di Luccio , J. A. Kornfield , A. Zak , R. Tenne , T. McNally , ACS Appl. Nano Mater. 2022, 5, 6385.

[25] L. Yadgarov , R. Tenne , Small 2024, 2400503. 10.1002/smll.202400503 PMC12272021 38953349

[26] H. Kwon , D. Bae , H. Jun , B. Ji , D. Won , J.‐H. Lee , Y.‐W. Son , H. Yang , S. Cho , Appl. Sci. 2020, 10, 3087 .

[27] N. Ling , S. Zheng , Y. Lee , M. Zhao , E. Kim , S. Cho , H. Yang , APL Mater. 2021, 9, 061108.

[28] J. Li , M. Hong , L. Sun , W. Zhang , H. Shu , H. Chang , ACS Appl. Mater. Interfaces 2018, 10, 458. 29235847 10.1021/acsami.7b13387

[29] M. S. Sokolikova , C. Mattevi , Chem. Soc. Rev. 2020, 49, 3952. 32452481 10.1039/d0cs00143k

[30] D. J. Hynek , E. Onder , J. L. Hart , G. Jin , M. Wang , R. M. Singhania , B. Davis , N. C. Strandwitz , J. J. Cha , Adv. Mater. Interfaces 2023, 10, 2202397.

[31] Y. Zhou , H. Jang , J. M. Woods , Y. Xie , P. Kumaravadivel , G. A. Pan , J. Liu , Y. Liu , D. G. Cahill , J. J. Cha , Adv. Funct. Mater. 2017, 27, 1605928.

[32] Y. Gong , Z. Lin , G. Ye , G. Shi , S. Feng , Y. Lei , A. L. Elias , N. Perea‐Lopez , R. Vajtai , H. Terrones , Z. Liu , M. Terrones , P. M. Ajayan , ACS Nano 2015, 9, 11658. 26502824 10.1021/acsnano.5b05594

[33] P. A. G. O'Hare , G. A. Hope , J. Chem. Thermodyn. 1992, 24, 639.

[34] M. Xu , B. Tang , Y. Lu , C. Zhu , Q. Lu , C. Zhu , L. Zheng , J. Zhang , N. Han , W. Fang , Y. Guo , J. Di , P. Song , Y. He , L. Kang , Z. Zhang , W. Zhao , C. Guan , X. Wang , Z. Liu , J. Am. Chem. Soc. 2021, 143, 18103. 34606266 10.1021/jacs.1c06786

[35] J. M. Woods , D. Hynek , P. Liu , M. Li , J. J. Cha , ACS Nano 2019, 13, 6455. 31141656 10.1021/acsnano.8b09342

[36] M. Hong , J. Li , W. Zhang , S. Liu , H. Chang , Energy Fuels 2018, 32, 6371.

[37] K. Ohnishi , M. Aoki , R. Ohshima , E. Shigematsu , Y. Ando , T. Takenobu , M. Shiraishi , Adv. Electron. Mater. 2023, 9, 2200647.

[38] R. Pickering , R. J. D. Tilley , J. Solid State Chem. 1976, 16, 247.

[39] J.‐H. Lin , Y.‐T. Yan , J.‐L. Qi , C.‐Y. Zha , Tungsten 2023, 6, 269.

[40] M. Juelsholt , A. S. Anker , T. L. Christiansen , M. R. V. Jørgensen , I. Kantor , D. R. Sørensen , K. M. Ø. Jensen , Nanoscale 2021, 13, 20144. 34846442 10.1039/d1nr05991b

[41] P. M. Rao , I. S. Cho , X. Zheng , Proc. Combust. Inst. 2013, 34, 2187.

[42] H. Kaper , I. Djerdj , S. Gross , H. Amenitsch , M. Antonietti , B. M. Smarsly , Phys. Chem. Chem. Phys. 2015, 17, 18138. 26102203 10.1039/c5cp01869b

[43] S. Bandi , A. K. Srivastav , CrystEngComm 2021, 23, 6559.

[44] L. Schlapbach , A. Züttel , Nature 2001, 414, 353. 11713542 10.1038/35104634

[45] Q. Li , C. He , Y. Wang , E. Liu , M. Wang , Y. Wang , J. Zeng , Z. Ma , T. Cao , C. Yi , N. Wang , K. Watanabe , T. Taniguchi , L. Shao , Y. Shi , X. Chen , S.‐J. Liang , Q.‐H. Wang , F. Miao , Nano Lett. 2018, 18, 7962. 30403355 10.1021/acs.nanolett.8b03924

[46] D. Chen , L. Gao , A. Yasumori , K. Kuroda , Y. Sugahara , Small 2008, 4, 1813. 18844301 10.1002/smll.200800205

[47] J. Yan , T. Wang , G. Wu , W. Dai , N. Guan , L. Li , J. Gong , Adv. Mater. 2015, 27, 1580. 25582656 10.1002/adma.201404792

[48] N. Kaur , D. Zappa , N. Poli , E. Comini , ACS Omega 2019, 4, 16336. 31616811 10.1021/acsomega.9b01792 PMC6787887

[49] Y. Kim , Y. I. Jhon , J. Park , J. H. Kim , S. Lee , Y. M. Jhon , Nanoscale 2016, 8, 2309. 26750205 10.1039/c5nr06098b

[50] K. Chen , Z. Chen , X. Wan , Z. Zheng , F. Xie , W. Chen , X. Gui , H. Chen , W. Xie , J. Xu , Adv. Mater. 2017, 29, 1700704. 10.1002/adma.201700704 28833622

[51] C. X. Zhao , S. Z. Deng , N. S. Xu , J. Chen , RSC Adv. 2015, 5, 70059.

[52] Y. H. Li , P. F. Liu , L. F. Pan , H. F. Wang , Z. Z. Yang , L. R. Zheng , P. Hu , H. J. Zhao , L. Gu , H. G. Yang , Nat. Commun. 2015, 6, 8064. 26286479 10.1038/ncomms9064 PMC4560788

[53] C.‐H. Lee , E. C. Silva , L. Calderin , M. A. T. Nguyen , M. J. Hollander , B. Bersch , T. E. Mallouk , J. A. Robinson , Sci. Rep. 2015, 5, 10013. 26066766 10.1038/srep10013 PMC5155493

[54] S. Zhao , L. Zhang , L. Deng , J. Ouyang , Q. Xu , X. Gao , Z. Zeng , Y.‐N. Liu , Small 2021, 17, 2103003. 10.1002/smll.202103003 34561966

[55] A. Ahad , Y. Yomogida , M. A. Rahman , A. Ihara , Y. Miyata , Y. Hirose , K. Shinokita , K. Matsuda , Z. Liu , K. Yanagi , Nano Lett. 2024, 24, 14286. 39404498 10.1021/acs.nanolett.4c03895

[56] Z. Liu , A. W. A. Murphy , C. Kuppe , D. C. Hooper , V. K. Valev , A. Ilie , ACS Nano 2019, 13, 3896. 30912636 10.1021/acsnano.8b06515 PMC7007277

[57] A. Alagh , F. E. Annanouch , A. Sierra‐Castillo , E. Haye , J.‐F. o. Colomer , E. Llobet , ACS Appl. Mater. Interfaces 2022, 14, 54946. 36469520 10.1021/acsami.2c16299 PMC9756288

[58] G. A. Asres , A. Dombovari , T. Sipola , R. Puskás , A. Kukovecz , Z. Kónya , A. Popov , J.‐F. Lin , G. S. Lorite , M. Mohl , G. Toth , A. Lloyd Spetz , K. Kordas , Sci. Rep. 2016, 6, 25610. 27180902 10.1038/srep25610 PMC4867582

[59] V. Kundrát , L. Novák , K. Bukvišová , J. Zálešák , E. Kolíbalová , R. Rosentsveig , M. B. Sreedhara , H. Shalom , L. Yadgarov , A. Zak , M. Kolíbal , R. Tenne , ACS Nano 2024, 18, 12284. 38698720 10.1021/acsnano.4c01150 PMC11100282

[60] A. Pelella , A. Kumar , K. Intonti , O. Durante , S. De Stefano , X. Han , Z. Li , Y. Guo , F. Giubileo , L. Camilli , M. Passacantando , A. Zak , A. Di Bartolomeo , Small 2024, 20, 2403965. 10.1002/smll.202403965 38994696

[61] M. Krause , A. Mücklich , A. Zak , G. Seifert , S. Gemming , Phys. Status Solidi B 2011, 248, 2716.

[62] T. Shinagawa , A. T. Garcia‐Esparza , K. Takanabe , Sci. Rep. 2015, 5, 13801. 26348156 10.1038/srep13801 PMC4642571

[63] H. Prats , K. Chan , Phys. Chem. Chem. Phys. 2021, 23, 27150. 34852033 10.1039/d1cp04134g

[64] D. Han , N. Gao , J. Ge , C. Liu , W. Xing , Catal. Commun. 2022, 164, 106427.

[65] T. Wu , M. Sun , H. H. Wong , C. H. Chan , L. Lu , Q. Lu , B. Chen , B. Huang , Inorg. Chem. Front. 2023, 10, 4632.

[66] X. Wang , J. Wang , B. Wei , N. Zhang , J. Xu , H. Miao , L. Liu , C. Su , Y. Li , Z. Wang , J. Mater. Sci. Technol. 2021, 78, 170.

[67] D. Xia , Z. Wang , S. Yang , Z. Cai , M. Hu , H. He , K. Zhou , Electrochim. Acta 2021, 385, 138409.

[68] H. Kwon , B. Ji , D. Bae , J.‐H. Lee , H. J. Park , D. H. Kim , Y.‐M. Kim , Y.‐W. Son , H. Yang , S. Cho , Appl. Surf. Sci. 2020, 515, 145972.

