# Supplementary material for: Active Sites‐Enriched Hierarchical Weyl Semimetal WTe2 Nanowire Arrays for Highly Efficient Hydrogen Evolution Reaction
Source: Adv Sci (Weinh). 2025 Apr 2;12(25):2500516. doi: 10.1002/advs.202500516 (PMC12224977; doi:10.1002/advs.202500516)
Supplement: Supplementary file 1 — Supporting Information [file ADVS-12-2500516-s001.docx]

Supporting Information

Active Sites-Enriched Hierarchical Weyl Semimetal WTe_2_ Nanowire Arrays for Highly Efficient Hydrogen Evolution Reaction

Hyeonkyeong Kim, Youngdong Yoo*

H. Kim

Department of Chemistry, Ajou University, Suwon 16499, Korea

Department of Energy Systems Research, Ajou University, Suwon 16499, Korea

Y. Yoo

Department of Chemistry, Ajou University, Suwon 16499, Korea

E-mail: yyoo@ajou.ac.kr

1. Experimental Section

1.1 Synthesis of WO_2.9_ NW arrays

WO_2.9_ nanowire (NW) arrays were synthesized directly on gold-deposited CC, quartz, silicon, sapphire, and mica substrates by reducing tungsten trioxide under high-temperature, low-pressure conditions. A layer of gold with a thickness of approximately 15–20 nm was deposited on the substrate using a sputtering method. An alumina boat containing 0.3 g of WO_3_ powder (Sigma-Aldrich, 99.5% purity) was positioned at the center of a 1-inch diameter quartz tube, while the substrate was placed 12 cm from the WO_3_ powder. After evacuating the quartz tube to a pressure of less than 6 mTorr, argon and hydrogen gases were introduced at flow rates of 100 standard cubic centimeters per minute (sccm) and 1 sccm, respectively. During the growth of the WO_2.9_ NWs, the pressure inside the tube was maintained at approximately 0.6 Torr. The WO_3_ powder was heated to 1,100°C at a rate of approximately 23.9°C per minute and held at that temperature for 60 min. Once the reaction was complete, the furnace lid was opened, and the furnace was allowed to cool to room temperature.

1.2 Synthesis of core–shell WO_3−x_–WTe_2_ NWs and WTe_2_ NWs

The synthesized WO_2.9_ NW arrays were enriched with Te to selectively form WTe_2_ NW arrays and core–shell WO_3−x_–WTe_2_ NW arrays. In a 1-inch diameter quartz tube, an alumina boat containing 0.3 g of Te slugs (Sigma-Aldrich, 99.999% purity) was positioned upstream, while the as-grown WO_2.9_ NWs/CC were placed downstream. After evacuating the quartz tube to less than 5 mTorr, argon gas was flowed at a rate of 400 sccm through a mass flow controller until the pressure inside the tube reached atmospheric pressure. Subsequently, both argon and hydrogen gases flowed at a rate of 20 sccm each. The WO_2.9_ NWs/CC were heated to 460°C at a rate of approximately 29°C min^−1^, while the Te slugs were heated to 50°C in 5 min and then raised to 550°C at a rate of approximately 33°C min^−1^. The selective synthesis of core–shell WO_3−x_–WTe_2_ NWs and WTe_2_ NWs was determined by the duration of tellurization. Core–shell WO_3−x_–WTe_2_ NW arrays were produced by holding the target temperature for 30 min, 1 h, or 2 h, while WTe_2_ NW arrays were synthesized by maintaining the target temperature for 4 h. Ten minutes after the reaction was completed, the furnace lid was opened, and the furnace was rapidly cooled to room temperature.

1.3 Characterization

SEM measurements of the synthesized NWs were performed on a JEOL JSM-6700F operating at 5 kV. XRD analysis was performed using a Rigaku Ultima III with a Cu Kα radiation point source (λ = 1.5406 Å). Raman spectroscopy was performed with 532 nm laser excitation, focused using a 100× objective lens. XPS measurements were performed on a Nexsa (Thermo Fisher Scientific) equipped with an Al Kα radiation source. The XPS spectra were calibrated using the carbon 1s peak at 284.8 eV. TEM and STEM images, as well as EDS maps, were acquired with a JEOL JEM-2100F. The absorption spectra of WO_2.9_ and WTe_2_ were collected using an EMC-11S-UV spectrophotometer.

1.4 Electrochemical measurements

All electrochemical measurements were performed using an SP-300 workstation at room temperature in a standard three-electrode electrochemical setup using a 0.5 H_2_SO_4_ (aq) electrolyte. A platinum wire served as the counter electrode, while an Ag/AgCl electrode acted as the reference electrode. The working electrodes comprised WTe_2_ NWs/CC, core–shell WO_3−x_–WTe_2_ NWs/CC, and WO_2.9_/CC, all grown on a conductive CC substrate. Polarization curves were recorded by sweeping the potential from 0 to −0.5 V (vs. RHE) at a scan rate of 10 mV/s. All potentials were converted to reversible hydrogen electrodes (RHE) using the equation: E_RHE_ = E_Ag/AgCl_ + 0.059 × pH + E^o^_Ag/AgCl_ (where E^o^_Ag/AgCl_ = 0.197 at 25°C). EIS was performed over a frequency range of 100 kHz to 0.01 Hz. The C_dl_ of the synthesized NWs was calculated from CV curves recorded at various scan rates ranging from 10 to 110 mV/s. To assess the stability of the catalyst, the WTe_2_ NWs arrays were subjected to measurements between 0.2 and −0.54 V vs. RHE at a scan rate of 10 mV/s for a total of 1,500 cycles.

**2 The HER mechanism in acidic medium**

The HER in acid electrolyte (2H^+^+2e^-^→H_2_) proceeds through the Volmer mechanism followed by the Tafel mechanism or the Heyrovsky mechanism.

Volmer reaction: H^+^ + e^-^ → H_ads_ + H_2_O

Tafel reaction: H_ads_ + H_ads_ → H_2_

Heyrovsky reaction: H_ads_ + H^+^ + e^-^ → H_2_ + H_2_O

H_ads_ denotes a hydrogen atom chemically adsorbed on the active site. Typically, the Tafel slopes of ~40, 30, and 120 mV/dec correspond to the rate-limiting steps for the Heyrovsky, Tafel, and Volmer reactions. Therefore, the synthesized WTe_2_ NWs with a 49mV/dec is determined by the Volmer-Heyrovsky mechanism as the rate-limiting step.


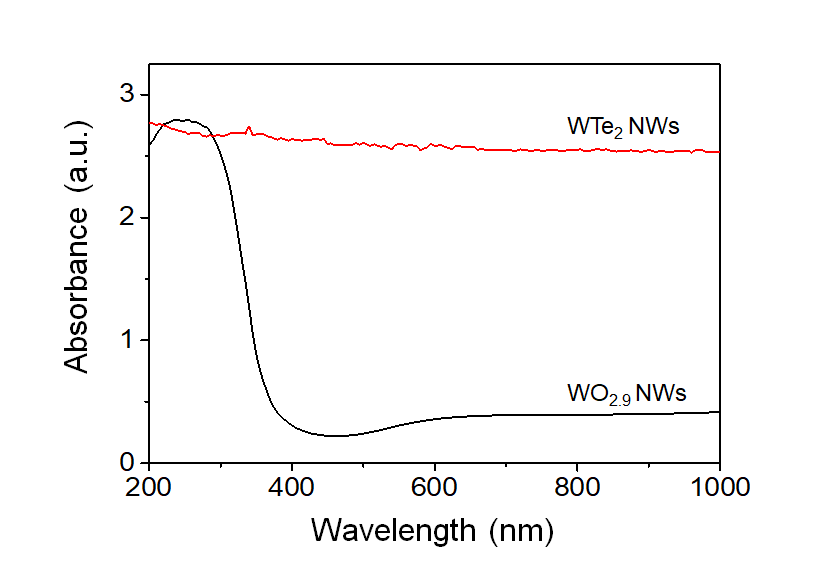


**Figure S1.** Ultraviolet-visible absorption spectra of the WO_2.9_ NWs and WTe_2_ NWs grown on quartz wafers.


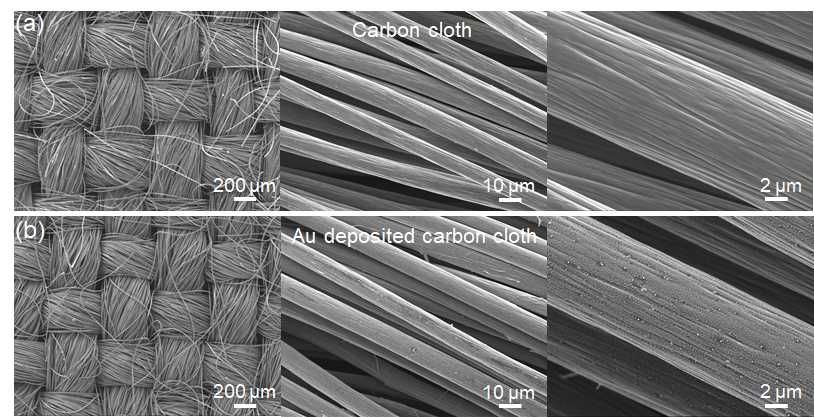


**Figure S2.** SEM images of (a) bare CC and (b) Au-deposited CC.


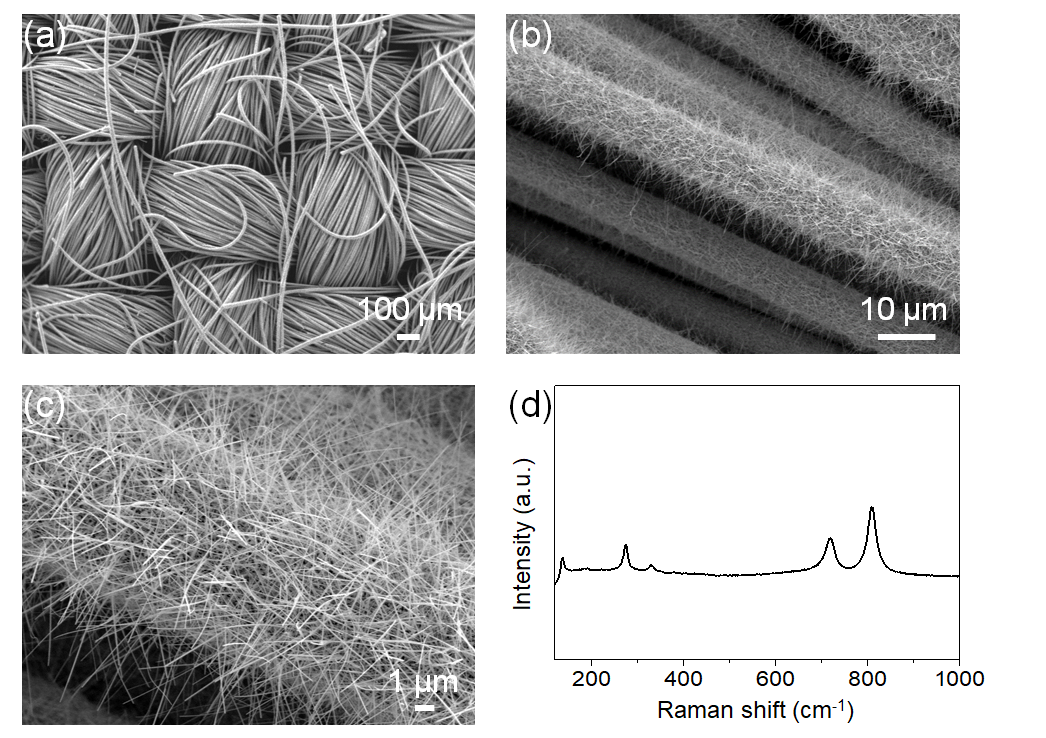


**Figure S3.** (a-c) SEM images and (d) Raman spectrum of WO_2.9_ NWs synthesized without Au nanoparticles.


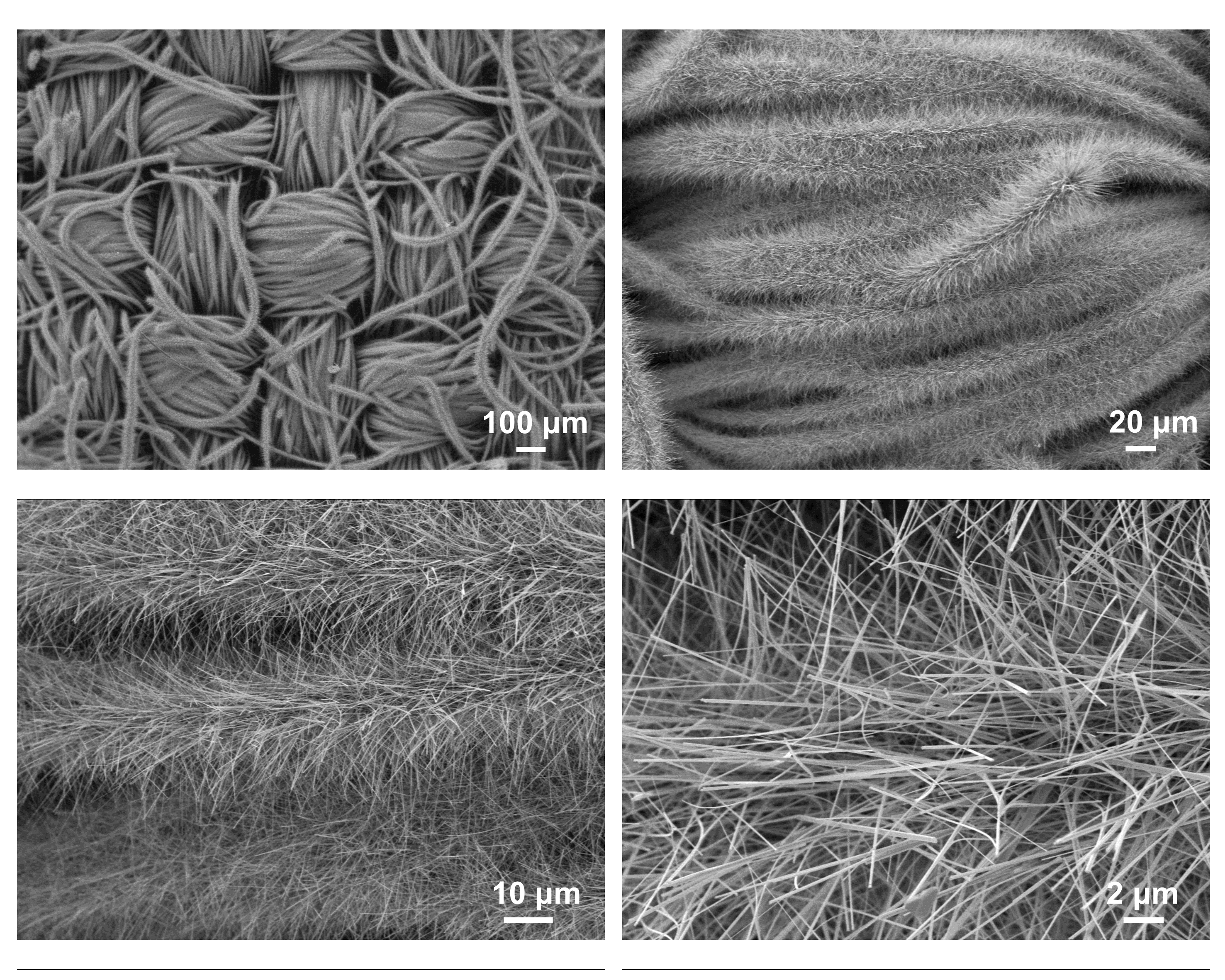


**Figure S4.** SEM images of core–shell WO_3−x_–WTe_2_ NWs (tellurization for 30 min).


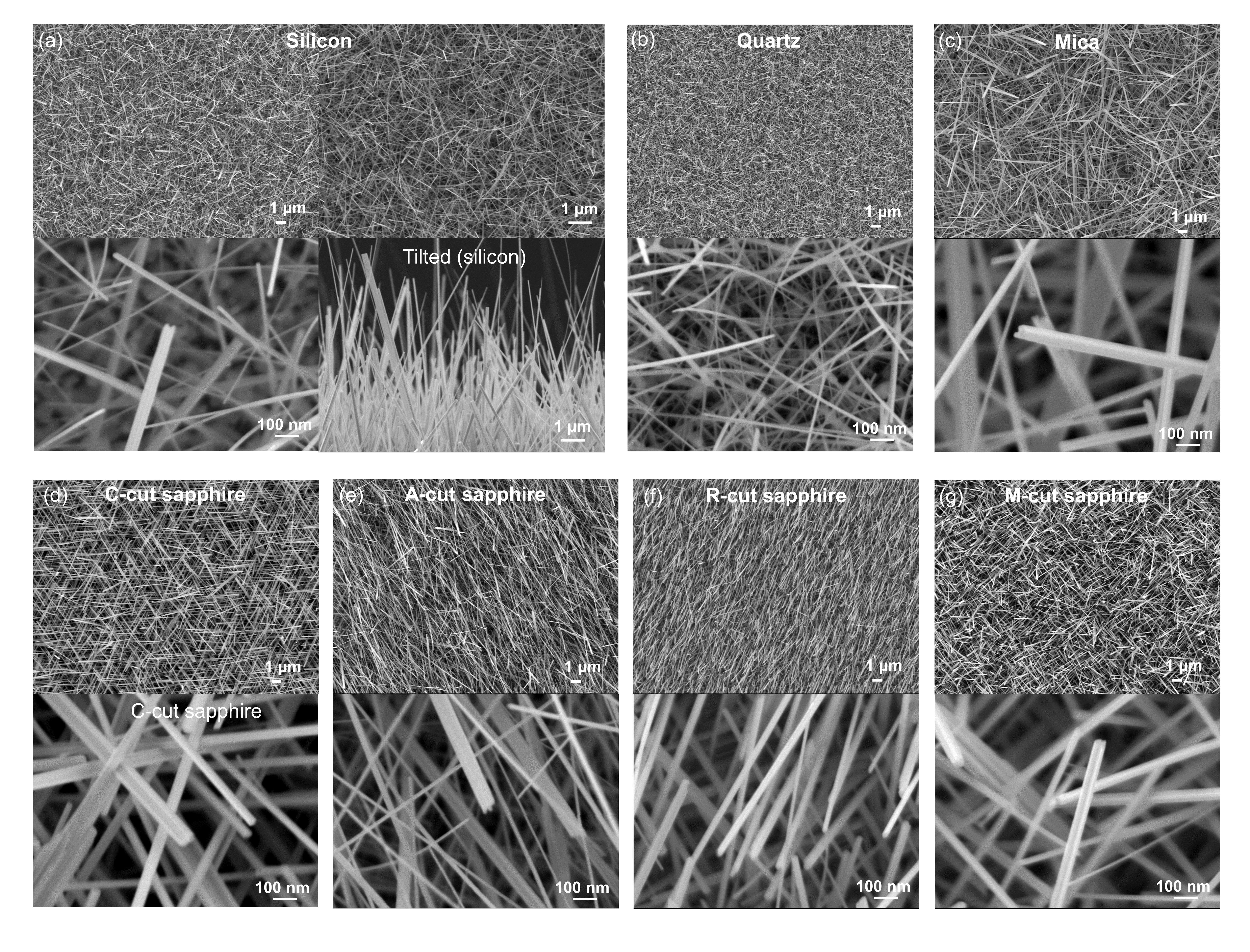


**Figure S5.** SEM images of as-synthesized WO_2.9_ NWs on the (a) silicon, (b) quartz, (c) mica, (d) c-cut sapphire, (e) a-cut sapphire, (f) r-cut sapphire, and (g) m-cut sapphire substrates.

**
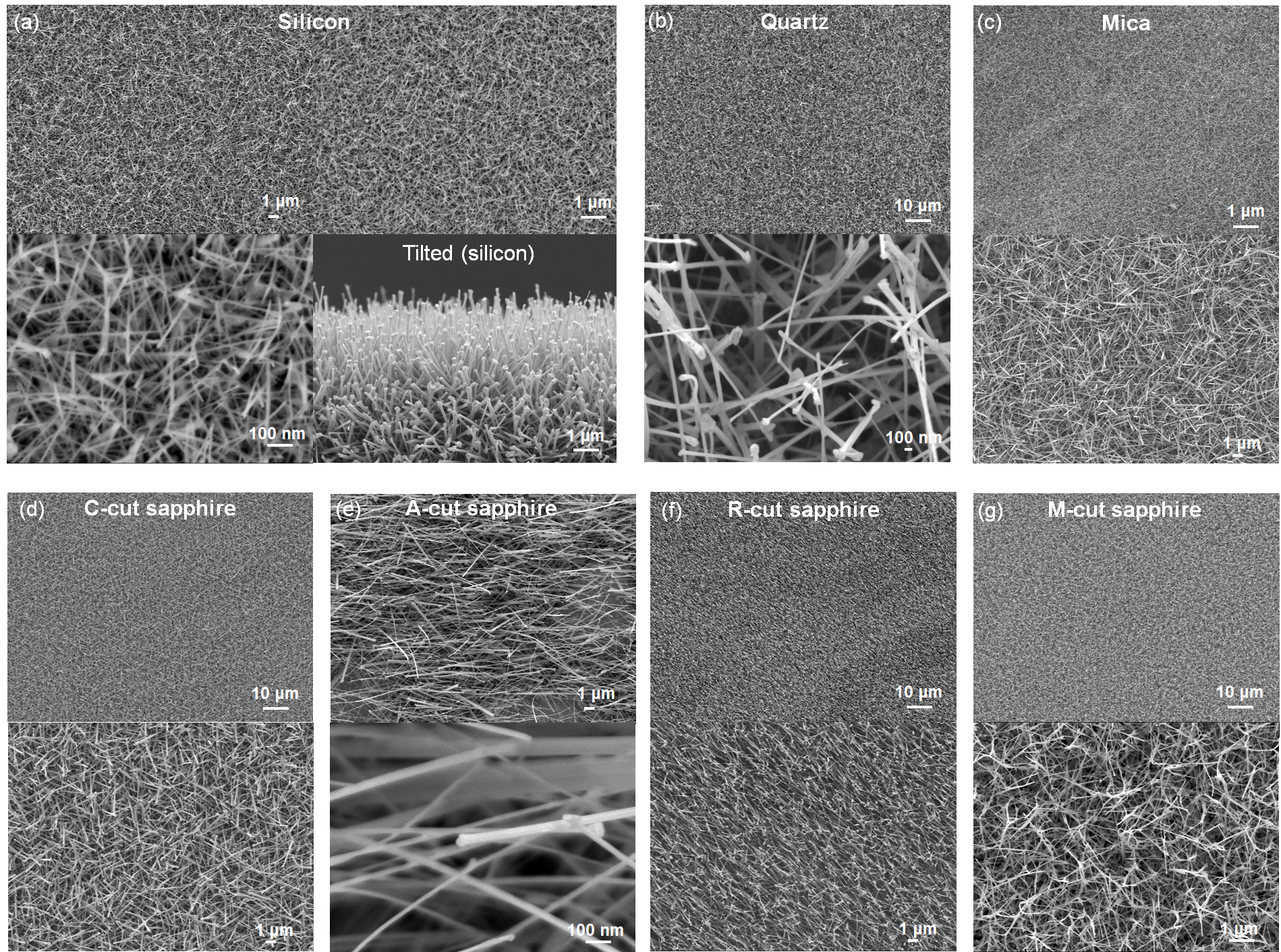
**

**Figure S6.** SEM images of as-synthesized WTe_2_ NWs on the (a) silicon, (b) quartz, (c) mica, (d) c-cut sapphire, (e) a-cut sapphire, (f) r-cut sapphire, and (g) m-cut sapphire substrates.


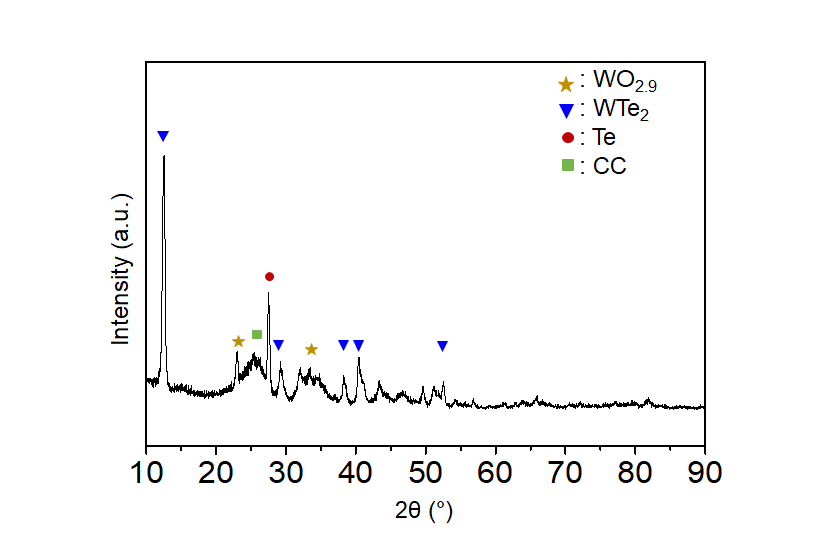


**Figure S7.** XRD patterns of core–shell WO_3−x_–WTe_2_ NWs obtained through the tellurization at low temperature (500°C) for 4 h.


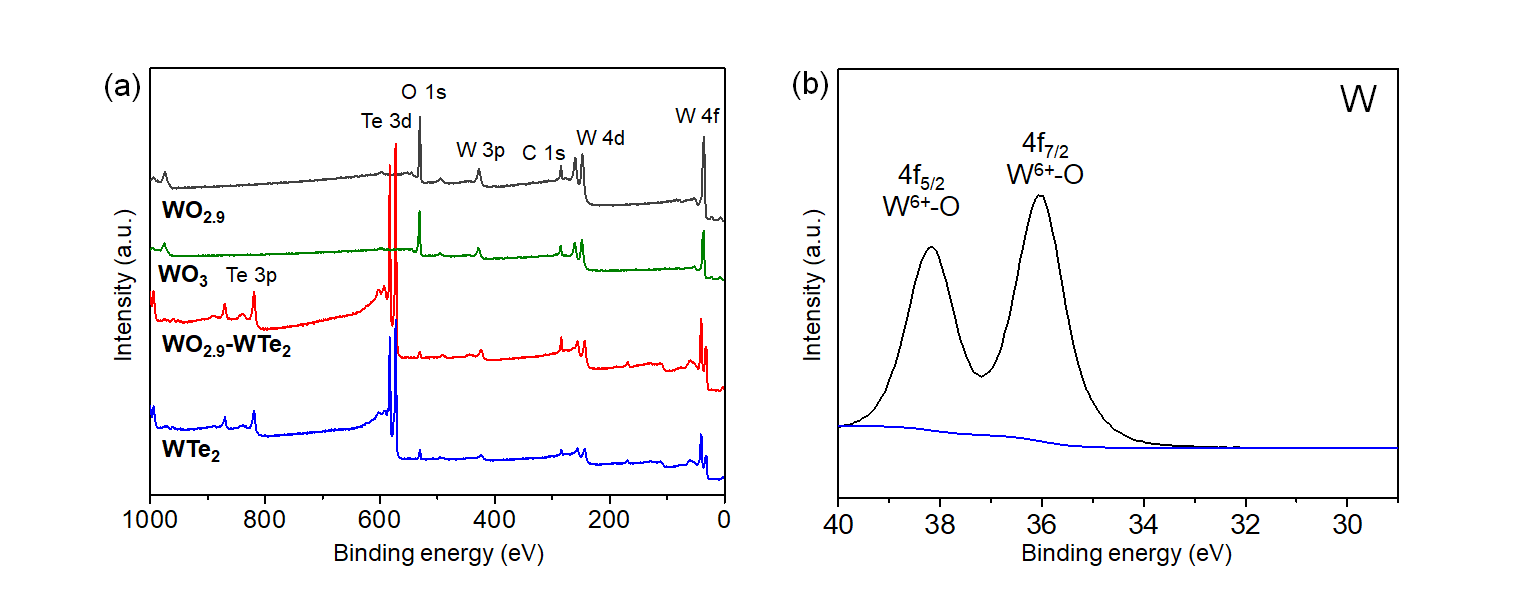


**Figure S8.** (a) XPS survey spectra of WO_2.9_ NWs, WO_3_ NWs, core–shell WO_3−x_–WTe_2_ NWs, WTe_2_ NWs. (b) XPS spectra for W 4f regions of the as-synthesized WO_3_ NWs.


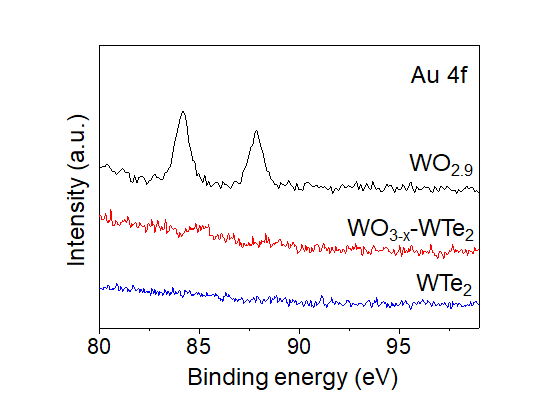


**Figure S9.** XPS spectra for Au 4f region of the WO_2.9_ NWs, core–shell WO_3−x_–WTe_2_ NWs (tellurization for 1 h), and WTe_2_ NWs.

**
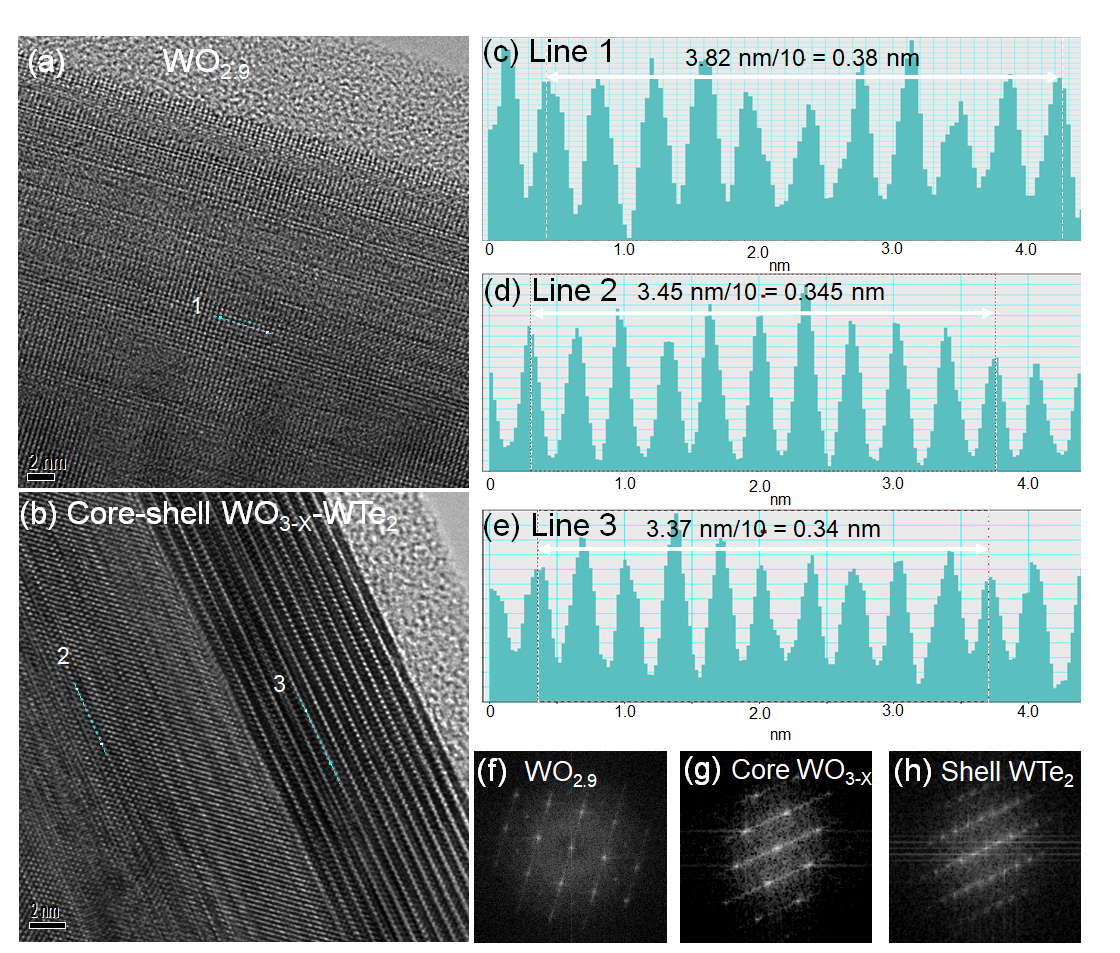
**

**Figure S10.** TEM images of (a) WO_2.9_, and (b) core–shell WO_3−x_–WTe_2_. Measuremets of lattice spacing with Gatan Digital Micrograph software of (c) line 1, (d) line 2, and (e) line 3 in (a) and (b). (f) FFT pattern of WO_2.9_ in (a). FFT patterns of (g) WO_3-x_ and (h) WTe_2_ in (b).


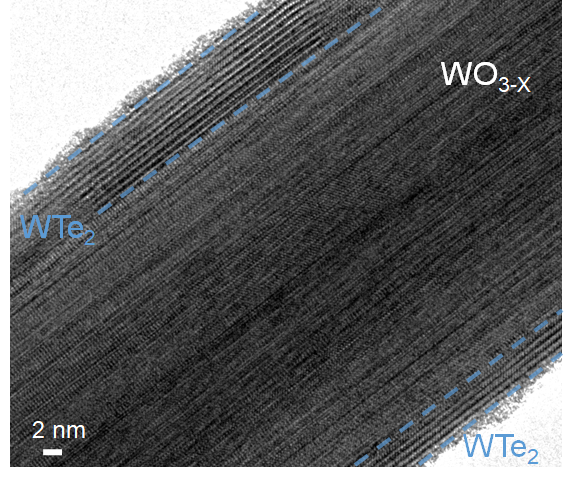


**Figure S11.** Low-magnification TEM image of the core–shell WO_3−x_–WTe_2_ NW.

**
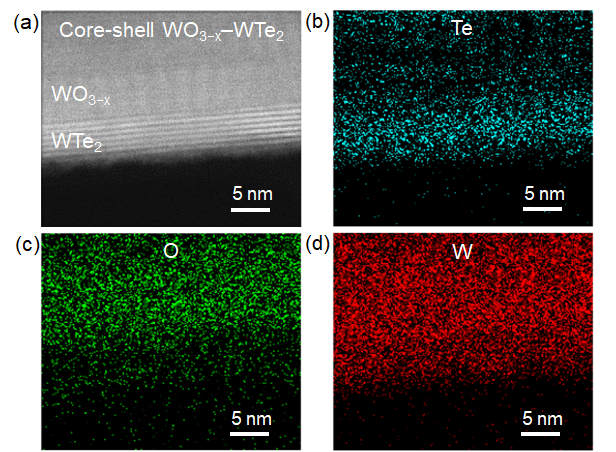
**

**Figure S12.** STEM image and EDS maps of a core–shell WO_3−x_–WTe_2_ NW (tellurization for 30 min). (a) STEM image of WO_3−x_–WTe_2_ NW. (b-d) EDS element maps for (b) Te, (c) O, and (d) W elements regions of WO_3−x_–WTe_2_.


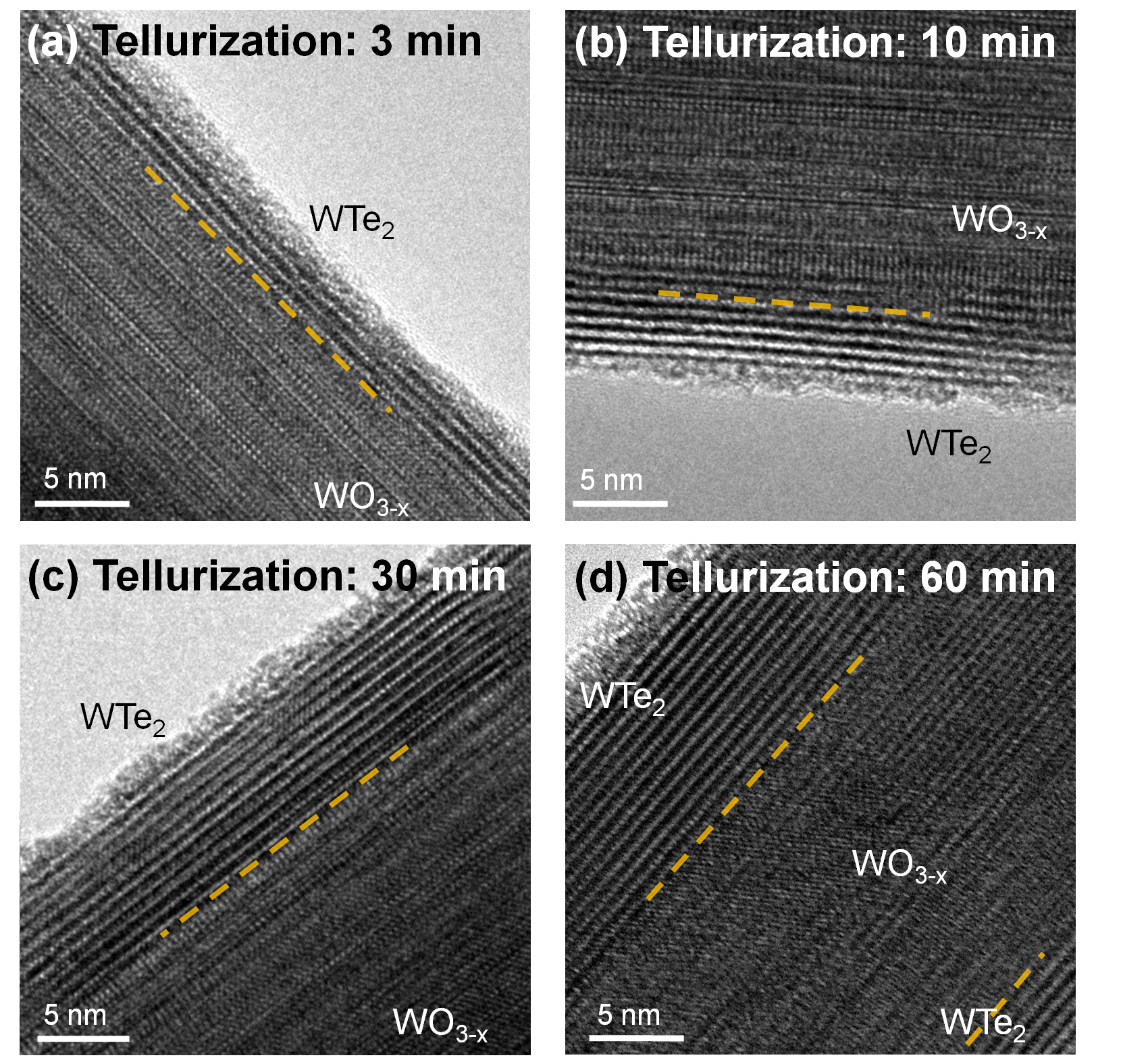


**Figure S13.** TEM images of the core–shell WO_3−x_–WTe_2_ NWs with different tellurization times. The TEM images confirm that the WTe_2_ shell of the core–shell WO_3-x_–WTe_2_ NWs thickens as the tellurization time increases. The core–shell WO_3-x_–WTe_2_ NWs obtained by tellurization of WO_2.9_ NWs for 3, 10, 30, and 60 min have WTe_2_ shells with thicknesses of approximately 3, 4.5, 8 and 10 nm, respectively.

**
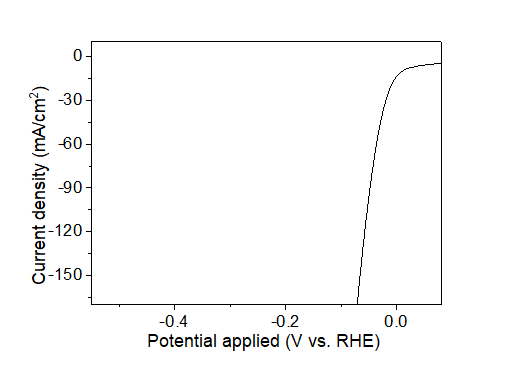
**

**Figure S14.** LSV polarization curve of the Pt/CC.


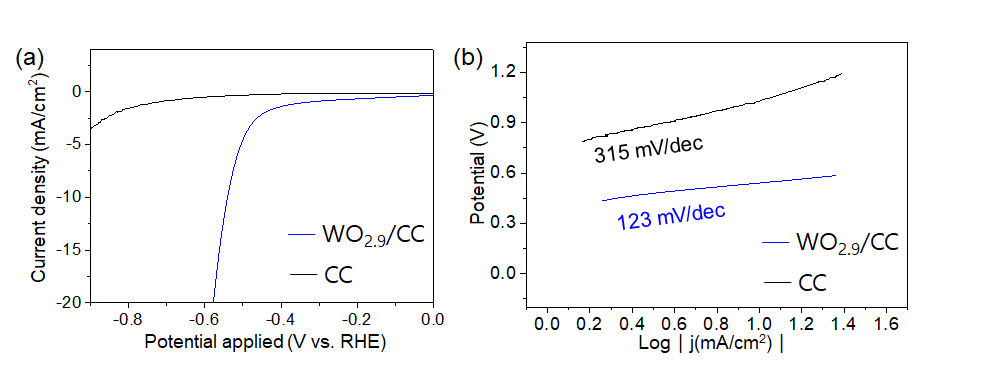


**Figure S15.** (a) LSV curves and (b) Tafel plots of the WO_2.9_/CC, and CC.

**Table S1.** Comparison of recent reported WTe_2_-based catalysts for HER.

| Catalysts | Synthesis method | Tafel slope  (mV dec^-1^) | η (V)  (at 10 mA cm^-2^) | Electrolyte | Ref |
| --- | --- | --- | --- | --- | --- |
| WTe_2_ NWs | Hydrogenation of WO_3_ and tellurization | 49 | 0.36 | 0.5 M H_2_SO_4_ | This work |
| WTe_2_ single crystals | The NaCl-Flux method | 169 | 0.692 | 0.5 M H_2_SO_4_ | [1]^]^ |
| WTe_2_ nanoribbons | Hydrothermal method and tellurization | 57 | 0.43 | 0.5 M H_2_SO_4_ | [2] |
| WTe_2_ nanosheets | Hydrothermal method and tellurization | 94.5 | 0.430 | 0.5 M H_2_SO_4_ | [3] |
| WTe_2_ by formation of amorphous phosphate nanoshells | Hydrothermal method, tellurization and conversion by phosphate | 77.9 | 0.220 | 0.5 M H_2_SO_4_ |  |
| WTe_2_ nanosheets | Hydrothermal method  and tellurization | 149 | 0.327 | 1.0 M KOH | [4] |
| Sulfur doped WTe_2_ nanosheets | Hydrothermal method, tellurization and sulfurization | 92 | 0.195 | 1.0 M KOH |  |
| WTe_2_ nanosheets | Chemical exfoliation | 381 | 0.708 | 0.5 M H_2_SO_4_ | [5] |
| Plasma-treated WTe_2_ nanosheets | Chemical exfoliation  and plasma treatment | 94 | 0.251 | 0.5 M H_2_SO_4_ |  |
| WTe_2_ single-crystals  (WTe_2_ after  8000 cylcle) | Mechanical exfoliation | 154 (79) | 0.707 (0.119) | 0.5 M H_2_SO_4_ | [6] |
| Te-deficient WTe_2_ | Mechanical exfoliation | 159 | 0.568 | 0.5 M H_2_SO_4_ |  |
| WTe_2_  (basal plane) | Mechanical exfoliation | 145 | 0.538 | 0.5 M H_2_SO_4_ | [7] |
| WTe_2_  (edge) | Mechanical exfoliation | 126 | 0.465 | 0.5 M H_2_SO_4_ |  |
| Plasma-treated WTe_2_ (basal plane) | Mechanical exfoliation | 105 | 0.407 | 0.5 M H_2_SO_4_ |  |
| Plasma-treated WTe_2_ (edge) | Mechanical exfoliation | 96 | 0.325 | 0.5 M H_2_SO_4_ |  |
| WTe_2_ flakes | Tellurization of a W  seed layer | 66 | 0.255 | 0.5 M H_2_SO_4_ | [8] |
| MoS_2_/WTe_2_ heterostructures | Mechanical exfoliation  and transfer | 40 | 0.140 | 0.5 M H_2_SO_4_ |  |

**Table S2.** Comparison of reported various morphologies of S- or Se- based TMD catalysts for HER.

| Catalysts | Tafel slope  (mV dec-1) | η | Electrolyte | Ref |
| --- | --- | --- | --- | --- |
| WS_2_ nanotubes | 113 | - | 1 M H_2_SO_4_ | [9] |
| WSe_2_ nanotubes | 99 | - | 1 M H_2_SO_4_ |  |
| WS_2(1–x)_Se_2_x nanotubes | 105 | - | 1 M H_2_SO_4_ |  |
| WS_2_ nanotubes | 115 | 0.355 V  at 10 mA cm^-2^ | 0.5 M H_2_SO_4_ | [10] |
| WS_2_ nanotriangles | 73 | 0.289 V  at 10 mA cm^-2^ | 0.5 M H_2_SO_4_ |  |
| MoS_2_ nanotubes | 84 | 0.223 V  at 10 mA cm^-2^ | 0.5 M H_2_SO_4_ |  |
| MoS_2_ nanoflowers | 76 | 0.239 V  at 10 mA cm^-2^ | 0.5 M H_2_SO_4_ |  |
| VS_2_ nanotubes | 154 | 0.588 V  at 10 mA cm^-2^ | 0.5 M H_2_SO_4_ |  |
| VS_2_ nanoflowers | 95 | 0.398 V  at 10 mA cm^-2^ | 0.5 M H_2_SO_4_ |  |
| WS_2_ nanotubes | 109 | 0.420 V  at 10 mA cm^-2^ | 0.5 M H_2_SO_4_ | [11] |
| MoS_2_ NWs | 90 | 0.35 V  at ∼2 mA cm^-2^ | 0.5 M H_2_SO_4_ | [12] |
| MoS_2_ particles | 134 | - | 0.5 M H_2_SO_4_ |  |
| WO_3_/WS_2_ core–shell nanorods | 174 | 0.20 V  at 5.5 mA cm^-2^ | 0.5 M H_2_SO_4_ | [13] |
| WO_3_ nanorods | 310 | 0.20 V  at 1.3 mA cm^-2^ | 0.5 M H_2_SO_4_ |  |


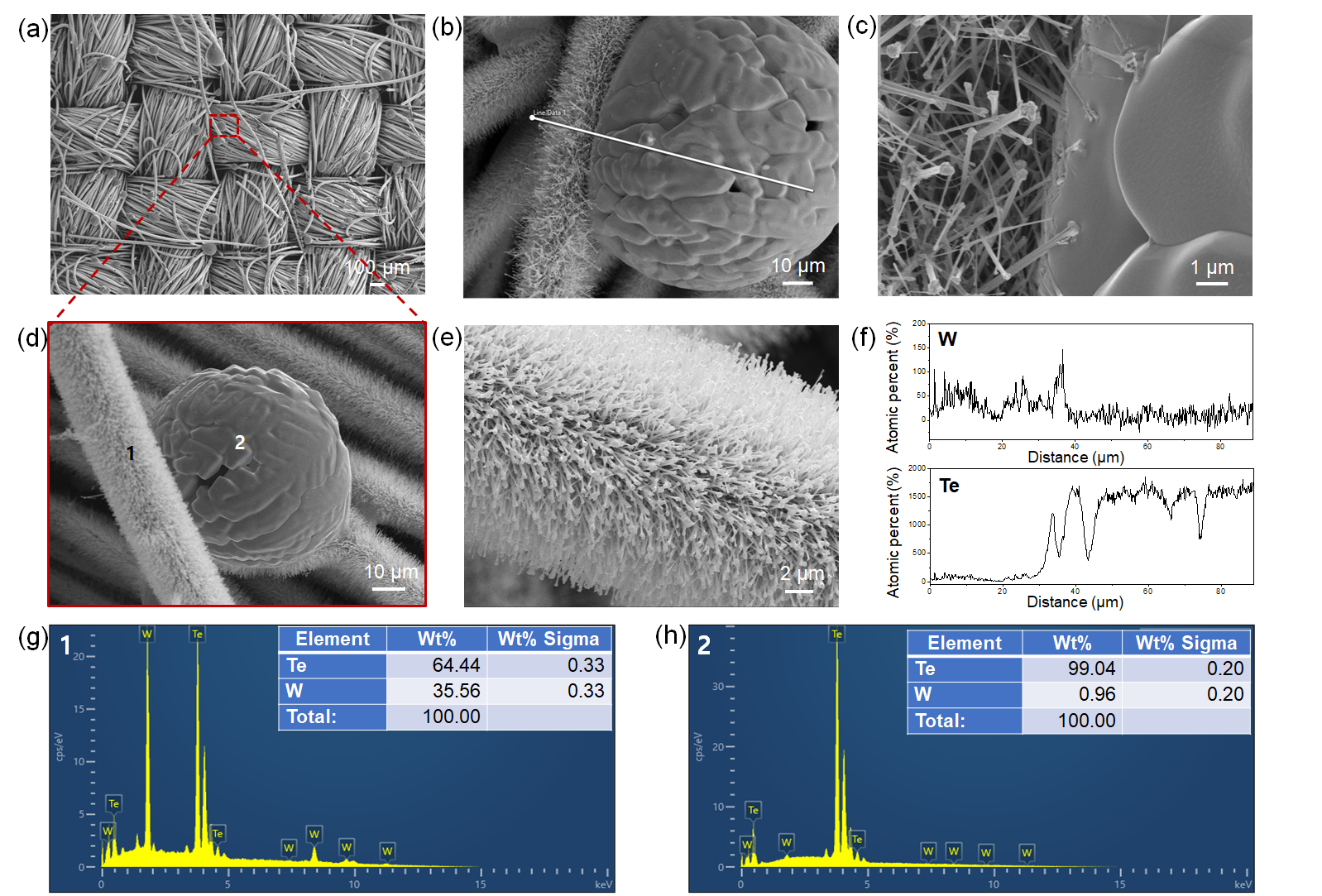


**Figure S16.** SEM images and EDS analysis of WTe_2_ NWs (tellurization for 6 h). (a-e) SEM images of the sample. (f) EDS lines taken obtained the line marked by (b). (g, h) EDS points in the regions marked by 1 and 2 in (d), respectively.


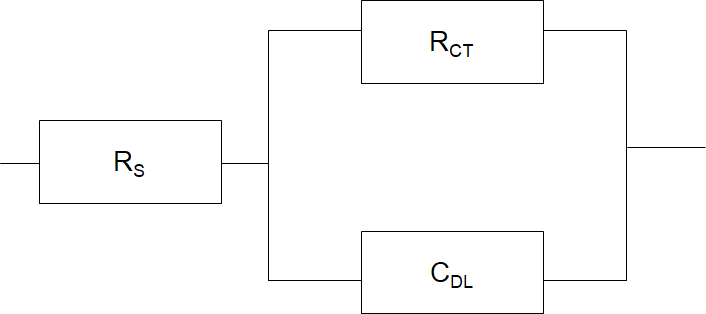


**Figure S17.** Depicts the equivalent RC circuit for te EC cell consisting of charge transfer resistance (R_CT_), serial resistance (R_S_), and electric double-layer capacitance (C_DL_).


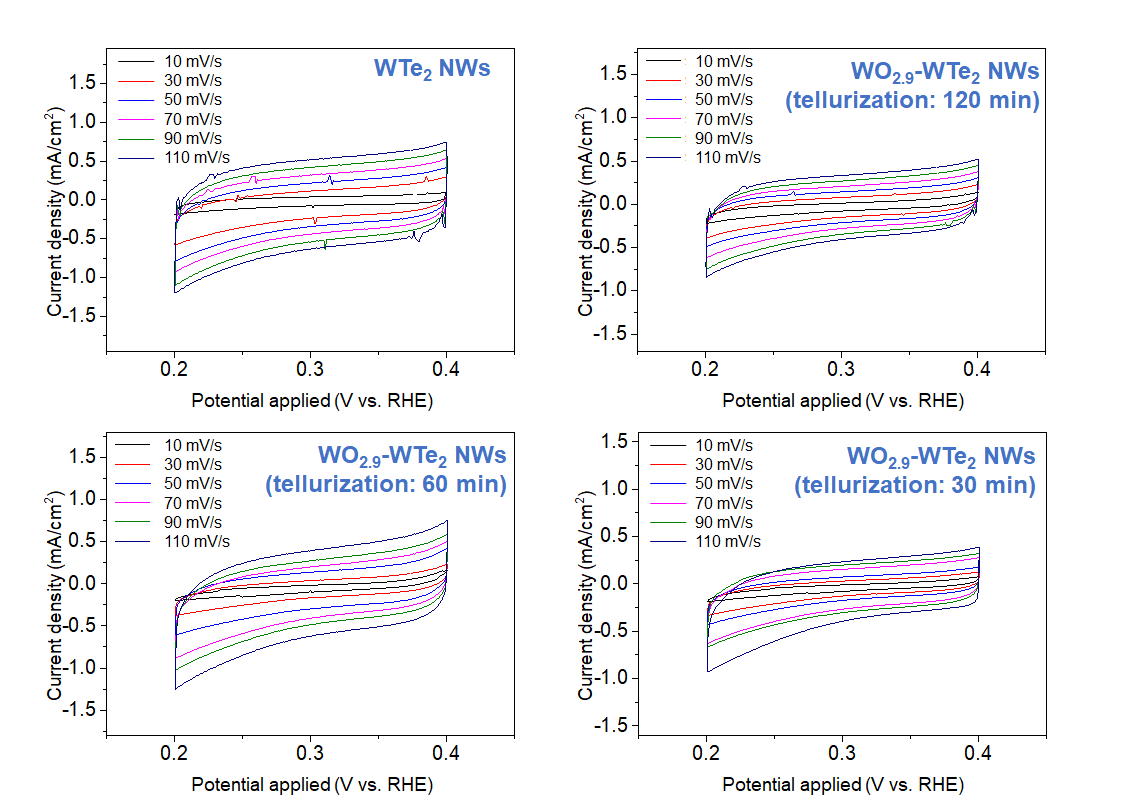


**Figure S18.** CV curves of WTe_2_ NWs/CC and core–shell WO_3−x_–WTe_2_ NWs/CC with different scan rates (10–110 mV/s).


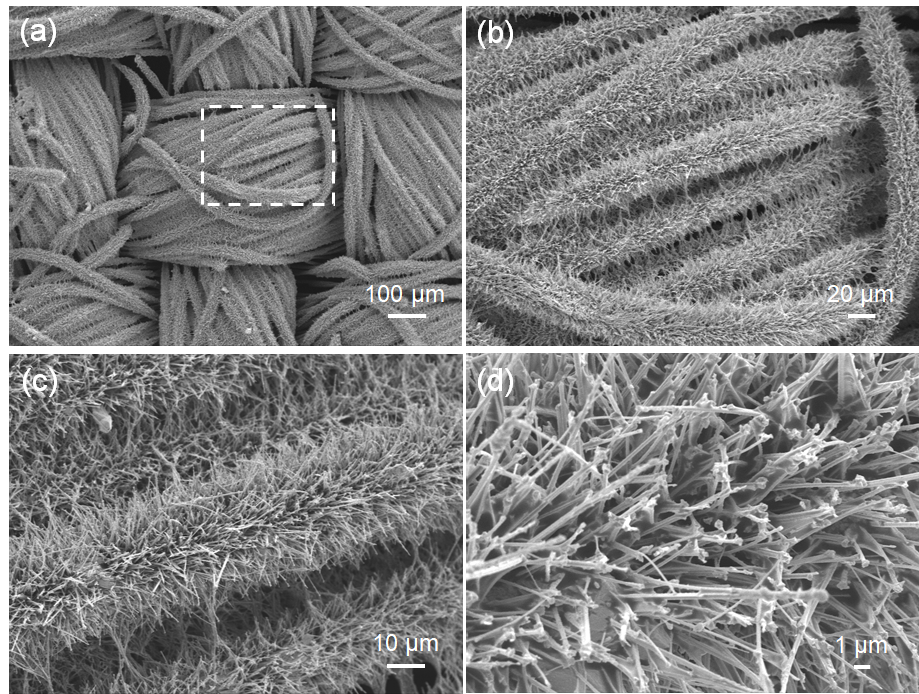


**Figure S19.** SEM images of WTe_2_ NWs/CC after 1,500 cycles.


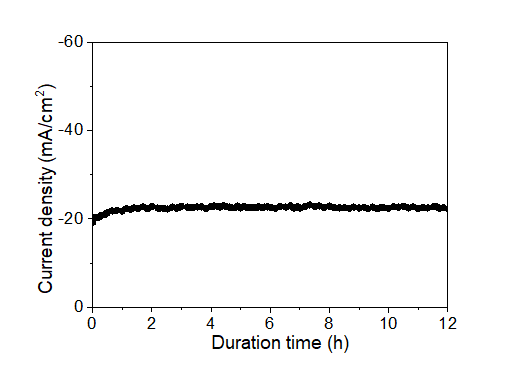


**Figure S20.** The stability test of WTe_2_ NW arrays at -0.38 V.

References

[1] H. Kwon, D. Bae, H. Jun, B. Ji, D. Won, J.-H. Lee, Y.-W. Son, H. Yang, S. Cho, *Applied Sciences*, 10.3390/app10093087.

[2] J. Li, M. Hong, L. Sun, W. Zhang, H. Shu, H. Chang, *ACS Applied Materials & Interfaces* **2018**, 10, 458.

[3] D. Xia, Z. Wang, S. Yang, Z. Cai, M. Hu, H. He, K. Zhou, *Electrochimica Acta* **2021**, 385, 138409.

[4] W. Lin, B. Zhang, J. Jiang, E. Liu, J. Sha, L. Ma, *ACS Applied Nano Materials* **2022**, 5, 7123.

[5] X. Wang, J. Wang, B. Wei, N. Zhang, J. Xu, H. Miao, L. Liu, C. Su, Y. Li, Z. Wang, *Journal of Materials Science & Technology* **2021**, 78, 170.

[6] H. Kwon, B. Ji, D. Bae, J.-H. Lee, H. J. Park, D. H. Kim, Y.-M. Kim, Y.-W. Son, H. Yang, S. Cho, *Applied Surface Science* **2020**, 515, 145972.

[7] N. Ling, S. Zheng, Y. Lee, M. Zhao, E. Kim, S. Cho, H. Yang, *APL Materials* **2021**, 9, 061108.

[8] Y. Zhou, J. V. Pondick, J. L. Silva, J. M. Woods, D. J. Hynek, G. Matthews, X. Shen, Q. Feng, W. Liu, Z. Lu, Z. Liang, B. Brena, Z. Cai, M. Wu, L. Jiao, S. Hu, H. Wang, C. M. Araujo, J. J. Cha, *Small* **2019**, 15, 1900078.

[9] K. Xu, F. Wang, Z. Wang, X. Zhan, Q. Wang, Z. Cheng, M. Safdar, J. He, ACS Nano 2014, 8, 8468.

[10] S. R. Kadam, M. Krishnappa, S. Ghosh, M. B. Sreedhara, A. Neyman, A. Upcher, E. Nativ Roth, L. Houben, A. Zak, A. N. Enyashin, R. Bar-Ziv, M. Bar-Sadan, Applied Materials Today 2024, 39, 102288.

[11] J. Lin, Z. Peng, G. Wang, D. Zakhidov, E. Larios, M. J. Yacaman, J. M. Tour, Advanced Energy Materials 2014, 4, 1301875.

[12] D. R. Cummins, U. Martinez, A. Sherehiy, R. Kappera, A. Martinez-Garcia, R. K. Schulze, J. Jasinski, J. Zhang, R. K. Gupta, J. Lou, M. Chhowalla, G. Sumanasekera, A. D. Mohite, M. K. Sunkara, G. Gupta, Nature Communications 2016, 7, 11857.

[13] P. Kumar, M. Singh, G. B. Reddy, ACS Applied Nano Materials 2019, 2, 1691.

[14] M. Kumar, T. C. Nagaiah, *Journal of Materials Chemistry A* **2023**, 11, 18336.

[15] M. Velpandian, A. Ragunathan, G. Ummethala, S. R. Krishna Malladi, P. Meduri, *ACS Applied Energy Materials* **2023**, 6, 5968.
